# Toward a Sustainable Energy Production System Based on Concentrated Solar Power Plants: Social and Water Availability Issues

**DOI:** 10.1021/acssuschemeng.5c11213

**Published:** 2026-02-12

**Authors:** Jose A. Luceño-Sanchez, Mariano Martin, Sandro Macchietto

**Affiliations:** † Department of Chemical Engineering, 4615 Imperial College London, South Kensington Campus, London SW7 2AZ, United Kingdom; ‡ Department of Chemical Engineering, 16779 University of Salamanca, Plaza de los Caídos 1-5, Salamanca 37008, Spain

**Keywords:** CSP, facility location, water-energy trade-off, mathematical optimization, MILP

## Abstract

The green transition of energy production systems is one of the most critical tasks for society nowadays. Concentrated solar power (CSP) plants require direct normal irradiance (*DNI*) to produce electricity. Nevertheless, the highest *DNI* values are usually found in regions with limited water availability, which can be a sustainability issue when using cooling technologies. Furthermore, deploying new infrastructure has significant socio-economic implications, requiring careful evaluation of CSP facility locations. In this work, a multiobjective mixed-integer linear programming model is developed, considering several variables related to production (such as *DNI*, temperature, and available land), environmental factors (such as water consumption by cooling systems), and social concerns associated with the deployment of the facilities. The model evaluates each region of Spain to choose the optimal location for five scenarios of technology substitution. The results show that decisions prioritize *DNI* values and seasonal energy demand followed by investment cost and social impact, while we avoid wet-cooling technology to increase job creation. Furthermore, a fully renewable electricity system using 9 CSP plants requires an estimated investment of 785 B€_2025_, presents a competitive LCOE of 0.086–0.093 €/kWh, and has the potential to contribute to a reduction in national unemployment by approximately 2.5 to 21%.

## Graphical abstract

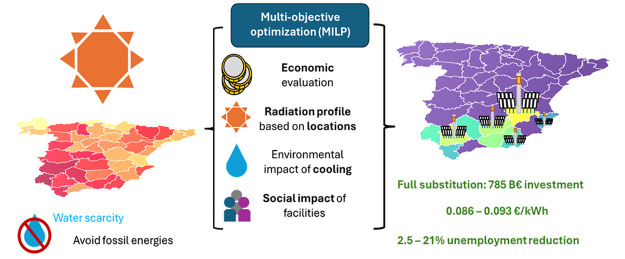

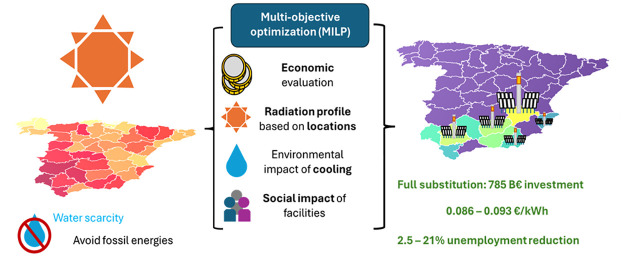

## 1 Introduction

Energy is among the most crucial resources in modern societies because it can be employed in a wide range of applications, from transportation to producing chemicals, and it can be related to countries’ development.^1,2^ For this reason, energy production and security are topics of concern, as seen in international treaties.^3^ In addition, the use of fossil fuels is decreasing in favor of renewable energies, such as wind and solar, toward a more sustainable system.

Solar energy is a renewable source of energy that can be used in any region of the Earth, even though the availability of solar irradiation is higher close to the Equator.^4^ This irradiation can be collected and transformed using two different technologies: photovoltaics and solar thermal plants. In solar thermal plants, or concentrated solar power (CSP) plants, solar irradiation heats a heat transfer fluid (HTF), which is used to generate a steam flow and produce electricity in a Rankine cycle, similar to traditional thermal power plants.^5,6^ The collection of solar irradiation can be done using four different technologies:^7^ (1) linear Fresnel reflectors, (2) parabolic trough collectors, (3) parabolic dish collectors, and (4) concentrating solar towers. Although there are several kinds of solar tower-based plants, depending on the type of solar receiver, they all present some significant trade-offs among the most important performance indicators: (1) solar irradiance, (2) water consumption, (3) social impact, and (4) capital cost. Higher solar irradiance is usually present in deserts or semiarid regions where water resources are scarce. In those locations, the traditional wet-cooling technologies can be replaced by dry-cooling systems, which do not depend on the water availability but require the consumption of a fraction of the power to operate the fans system, reducing the net power production of the facility^8^; this use of water to generate electricity and the use of electricity to treat and pump water generate a trade-off usually called a “water–energy nexus”,^9^ which represents a notable concern nowadays due to the potential environmental impact of the facility. Furthermore, regarding the possible related social implications, the facility’s location can be more beneficial for a specific region than others.

CSP plants have been studied using a wide range of approaches. From a mathematical formulation perspective, the studies have assessed water consumption for plant operation and cooling,^10^ as well as to support the design of key equipment, such as the cooling system^11^ or the heat exchanger network.^12^ In terms of technology evaluation, comparative studies have evaluated the cost-effectiveness of various CSP technologies, including solar towers and parabolic troughs, under different regional conditions.^13^ Regarding economic evaluation, LCOE estimations were carried out both with equation-based methods and also with genetic algorithms.^14^ For plant deployment, geospatial techniques such as GIS and multicriteria decision making have been applied to identify the most suitable locations, taking into account factors such as solar irradiance^15^ or proximity to electrical infrastructure.^16^ Finally, integrating CSP with other applications, such as desalination,^17^ biomass, or agriculture,^18^ has been investigated as a way to enhance system sustainability. Despite these efforts, a comprehensive analysis that simultaneously considers incident radiation, social impact, investment costs, and the water-energy nexus for a specific location is still missing. Conducting such an integrated study could support better-informed decisions and contribute to the development of more sustainable and efficient CSP plants.

In this work, a multiobjective optimization formulation is developed to study the influence of different key design variables (i.e., number of facilities, installed power capacity, cooling technology, etc.) and regional data (i.e., *DNI*, sun hours, population, GDP, etc.) on the location and distribution of facilities across a country in order to produce a model suitable for strategic decision-making in CSP facility deployment. The work is organized into five sections: the first section covers the model formulation of the problem; the second section shows the optimization procedure; the third section presents the case study considered, giving the values of specific location variables; in the fourth section, the results are shown and discussed; and finally, conclusions are drawn.

## 2 Model Formulation

The structure of CSP power plants can involve different technological solutions for each section, mainly a solar radiation capture system, energy storage, thermal cycle, and cooling. In the case of solar radiation capture systems, the solar tower option is the technology with the highest energy production potential, even though it is not as developed as parabolic troughs.^19^ The available energy storage solutions cover water/steam to molten salts, heat transfer oils, or liquid metals: liquid metals present corrosion and safety problems at high temperature, heat transfer oils have lower thermal performances than other solutions, and the heat storage capacity of water/steam is lower than molten salts one^20^; thus, molten salts are more employed. Nevertheless, the operation conditions have to be controlled to avoid corrosion. Regarding the thermal cycle, there are two main options for CSP plants: the Brayton cycle (He or CO_2_) and Rankine cycle (Steam or CO_2_).^21^ As the Rankine steam cycle can achieve high thermal efficiency at high temperatures and is a commercial mature technology,^22^ it is often employed in CSP plants^23^; furthermore, the efficiency of the cycle can be improved applying additional steps such as superheating and reheating. Thus, the architecture of a CSP power plant considered in this work consists of a solar tower/central tubular receiver, molten salts as energy storage, and a regenerative Rankine cycle with superheating and reheating (RRC). Figure 1 shows the scheme of the CSP plant studied.

**Figure 1:**
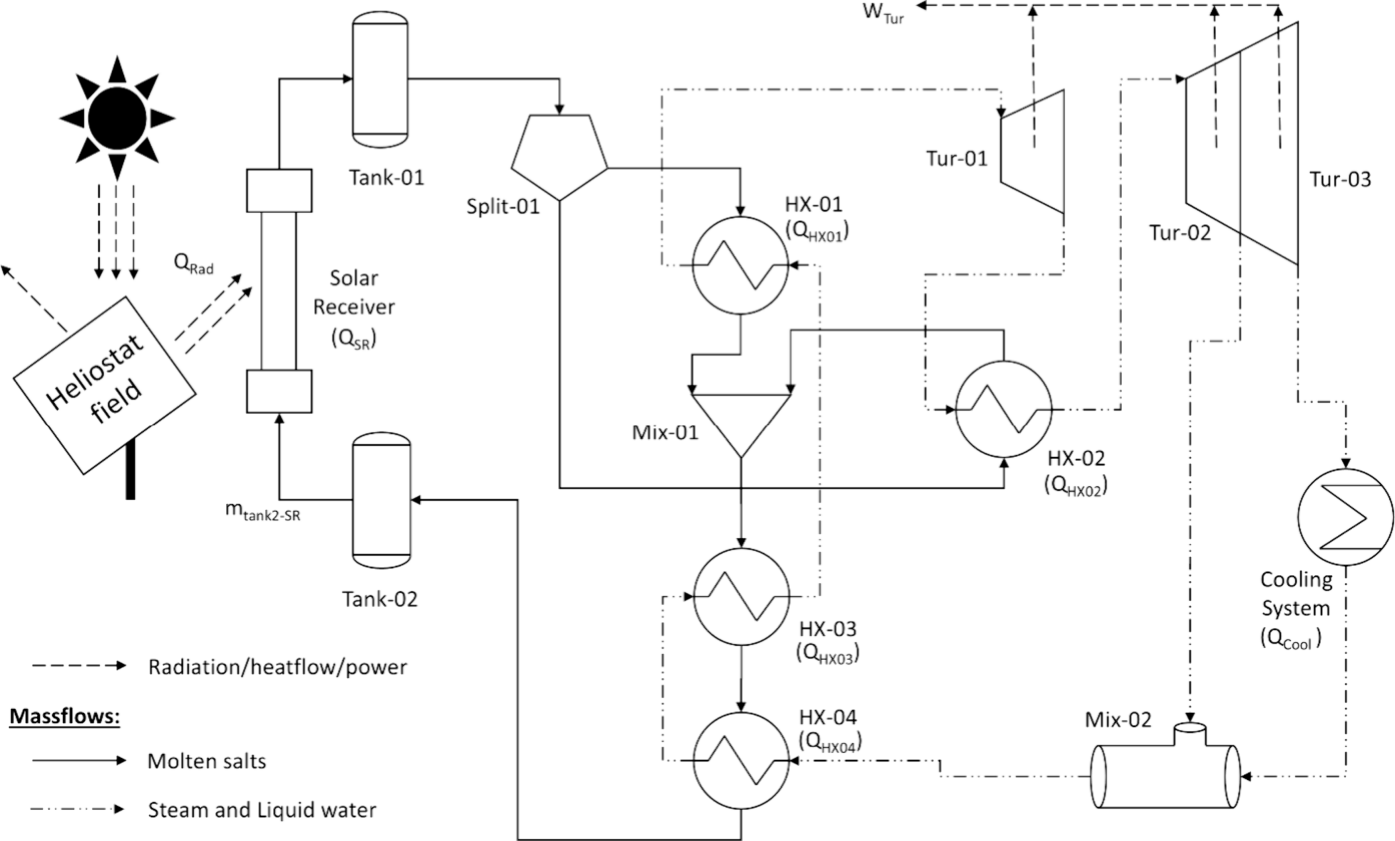
Regenerative Rankine cycle with superheat and reheat applied in a CSP plant. Heat flows (*Q*) and turbine power (W) are labeled in the corresponding equipment.

The facility can be divided into three different sections: (1) the solar field, (2) the thermal cycle, and (3) the cooling system. The facility can be modeled using different tools, such as modular third-party software^24^ or equation-based frameworks^25^ and discretization techniques, i.e., modeling each section or equipment separately, section per section, or the entire plant as one.

The modeling was carried out in 4 different steps: (1) a detailed model from previous works^26^ was applied to evaluate the performance of the facility (see Supporting Information Section S1); (2) a surrogate model is developed for the facility based on input–output ratios from the detailed model; (3) with the results of the previous step, the mass and heat flow rates of each stream are determined and employed to obtain numerical relations between the variables of interest, such as the ratio between the energy produced and the inlet stream, using data from previous work^25^; and (4) a facility location problem is formulated using a surrogate model of the facility. This approach reduces the complexity of the facility location problem but also maintains the essence of the solar thermal power plants because the location variables are considered out of thermal cycle operation.

This work does not consider the possible existence of multiple facilities in a particular location. Thus, there is a maximum of one facility per location. The total number of facilities (*n*_*fac*_) is distributed across all possible locations: each facility can be located in a different location, and the time horizon is included. To formulate the model, two sets of variables are introduced to reduce the complexity of the nomenclature:

1. Number of time discretization periods *TD* = {1,2, ···, *n*_*TD*_}. This set defines the number of time points considered in the study, and *t* represents the period in which the variables are being evaluated, assuming that *t* ∈ TD.
2. Number of locations *Loc* = {1,2, ···, *n*_*Loc*_}. It corresponds to the possible locations for the facilities. The index of this set is denoted as *l* ∈ Loc.

Given the actual electricity demand to be substituted, the meteorological data (*DNI*, temperature, humidity, etc.), economic-social data (population density, GDP, and unemployment) of the regions across a country, and the key performance indicators of a CSP facility, we minimize an aggregated objective to decide on the location and size of the plants. This evaluation is performed through an optimization that minimizes the objective function presented in Section 3.2, jointly assessing electricity production as well as economic, social, and environmental impacts.

### 2.1 Surrogate Model of the CSP plant

Considering that no chemical reactions occur in any equipment, most CSP plant equations behave linearly with the power input (Supporting Information Section S1). Thermodynamic properties (e.g., *C*_*p*_) remain unchanged, and operating temperatures and pressures are intensive variables that are typically fixed. Therefore, water and steam mass flow rates scale linearly with the turbine power output. Design features, such as the number of heliostats, remain constant, while operating parameters, such as monthly sun hours, vary but preserve the linearity of the equations. These proportional relations (*PK*) are expressed as the ratio between a variable (*Variable*) and a reference (*Reference*), such as the incoming radiation and the power supplied by the turbines, as seen in eq 1. In this work, the eight variable relations considered are presented in Table 1.

$$\frac{Variable}{Reference}={PK}_{number}$$ 1

**Table 1:** Relations between the Interest Variables

**number**	**variable**	**reference**	***PK* value**	**unit of *PK***
1	*Q_SR_*	*Q_Rad_*	3.2883 × 10^–3^	kW/kW
2	*m_tank_*_2_ *_‑SR_*	*Q_SR_*	2.3981 × 10^–3^	(kg/s)/kW
3	*W_Turb_*	*m_tank_*_1_ *_‑split_*_1_	168.4195	kW /(kg/s)
4	*Q_cool_*	*m_tank_*_1_ *_‑split_*_1_	–248.6300	kW /(kg/s)
5	*Q_HX_*_1_	*m_tank_*_1_ *_‑split_*_1_	103.9966	kW /(kg/s)
6	*Q_HX_*_2_	*m_tank_*_1_ *_‑split_*_1_	147.8651	kW /(kg/s)
7	*Q_HX_*_3_	*m_tank_*_1_ *_‑split_*_1_	93.9388	kW /(kg/s)
8	*Q_HX_*_4_	*m_tank_*_1_ *_‑split_*_1_	71.2449	kW /(kg/s)

where *Q*_*SR*_ and *Q*_*Rad*_ are the solar receiver incoming heat flow and the radiation heat flow from the solar field (kW), *m*_*tank*2‑*SR*_ and *m*_*tank*1‑*split*1_ are the mass flow rate from storage tank 2 to the solar receiver and the mass flow rate that leaves storage tank 1 to splitter 1 (kg/s), *W*_*Turb*_ is the power produced by the turbine system (kW), *Q*_*cool*_ is the cooling requirements (kW), and *Q*_*HX*1_–*Q*_*HX*4_ are the heat flow in each heat exchanger (kW). This formulation presents the advantage of expressing every desired variable as a function of the solar radiation income, which is the main parameter in CSP plant conceptual design because it is the “raw material”.

Regarding radiation, it was studied using direct normal irradiance (*DNI*, kWh/(m^2^·K)) as the variable to assess the input solar radiation at a specific location. This variable is widely employed in solar applications.^27,28^

The radiation heat flow (*Q_R__ad_*, kW) that the HTF will absorb can be determined considering 3 inputs: (1) the incoming radiation, i.e., *DNI*; (2) the daily operation time considered, which is *H*_*sun*_ because the solar receiver only operates when there is sunlight; and (3) the studied area, which is considered as the total effective heliostat area. The required value of *Q*_*Rad*_ for a location *l* is determined by eq 2. It is important to note that this variable is also related to *Q*_*SR*_, which can be calculated using eq 3 according to *PK*_1_ definition (see Table 1):

$$Q_{Rad,t,l}=\frac{{\mathrm{DNI}}_{t,l}\cdot A_{hel}\cdot n_{hel,l}\cdot \eta_{hel}}{H_{sun,l,t}}\forall t\in TD,\forall l\in Loc$$ 2

$$Q_{Rad,t,l}\cdot {PK}_{1}=Q_{SR,t,l}\forall l\in Loc$$ 3

where *A*_*hel*_ is the area of a heliostat (m^2^), *n*_*hel*_ is the number of heliostats in the *l* location, and η_*hel*_ is the heliostat field efficiency. Usually, the value of η_*hel*_ is determined considering the heliostat and the field individual efficiencies^25,29^ ; η_*hel*_ was assumed to be 90%, and the typical global field efficiency (55%) is included in the values of *PK*_1_. The value of *A*_*hel*_ is specified by manufacturers, which is considered as 120 m^2^/heliostat^25^; furthermore, the footprint related to a heliostat was regarded as the same value as *A*_*hel*_. This footprint presents a constraint related to ground availability (*Ground_ava_*): only a small fraction of the total surface can be employed for industrial applications. It is also important to note that the large shape of the facility layout area (*A*_layout_) is related to the heliostat field footprint; thus, the product of *A*_*hel*_ and *n*_*hel*_ is considered to include the total surface covered by the facility. Thus, a maximum of 0.5% (*Ground_ava_* = 0.005) of the province total area (*A*_*Prov*_) is assumed for *A*_layout_, as shown in eq. 4:

$$A_{hel}\cdot n_{hel,l}\approx A_{layout,l}\leq A_{Prov,l}\cdot {Ground}_{ava}\forall l\in Loc$$ 4

After determining *Q*_*SR*_, the mass flow rate *m*_*tank*2‑SR_ is calculated using eq 5. The mass flow rate *m*_*tank*1‑*split*1_ can be determined knowing *m*_*tank*2‑*SR*_, but considering the dependence with the sun hours of the facility location (*H*_*sun*_, h), as seen in eq 6:

$$m_{tank2-SR,t,l}=Q_{SR,t,l}\cdot {PK}_{2}\forall t\in TD,\forall l\in Loc$$ 5

$$m_{\tan k1-\mathrm{split}1,t,l}=m_{\tan k2-\mathrm{SR},t,l}\cdot \left(\frac{H_{\mathrm{sun},t,l}}{24}\right)\forall t\in \mathrm{TD},\forall l\in \mathrm{Loc}$$ 6

Due to the power consumption related to critical operations (e.g., molten salt pumping and increment of water pressure) being much lower than 3% of the reference model,^26^ it is assumed that these power losses are included in the calculation of the net power of the turbine system, represented in relation *PK*_3_.

### 2.2 Power Production, Cooling Requirements, and Heat Flows

In any country, a minimum power demand (*W*_*Dem*_, kW) must be met for each *t* ∈ *TD* considered. In a CSP system, *W*_*Dem*_ can be achieved considering the power balance shown in eq. 7:

$$W_{Dem,t}\leq \sum_{l=1}^{Loc}W_{net,t,l}+W_{add,t}\forall t\in TD$$ 7

There are two contributions considered in eq 7: (1) the net power production that the set of facilities will provide (*W*_*net*_, kW), determined mainly by the location due to the effect of *DNI* and the facility size; and (2) *W*_*add*,*t*_, the additional power supply produced using other sources to fulfill the power demand (kW), in the case where the demand could not be met by the facilities, which implies an additional penalty/purchase cost.

The total number of facilities (*n*_*fac*_) could be lower than the maximum value (*n*_*fac*_^*max*^) because larger facilities can be built to meet the production demand and advantage economies of scale if the area is available. In this work, it is assumed that only one facility can be deployed in each location, as the objective is to study the cumulative power capacity. Thus, a new binary variable *y*_*ff*_ is defined to indicate whether a facility exists in location *l*, as presented in eq 8.

$$\sum_{l=1}^{Loc}y_{ff,l}=n_{fac}$$ 8

The value of *W*_*net*_ is not the same as the power produced by the turbine system of the facility (*W*_*Turb*_, kW) because the cooling system installed may require electricity. There are two main cooling technologies for CSP plants: wet-cooling (WC) and dry-cooling (DC). The presence of one or the other system is linked beforehand by the existence of the facility in this location, as seen in eq 9, where *y*_*DC*_ is the binary variable of DC existence and *y*_*WC*_ is the binary variable of WC existence.

$$y_{DC,l}+y_{WC,l}=y_{ff,l}\forall l\in Loc$$ 9

*W*_*net*_ is calculated using eq 10. In the case of wet-cooling, *W*_*net*_ has the same value as *W*_*Turb*_ but, if there is dry-cooling instead, the value of *W*_*net*_ will be lower due to the power consumption of the fans. *Cons*_*DC*_ is the ratio between the power consumption of the dry-cooling fans system to operate them and the power produced by the plant (%). The value *Cons*_*DC*_ can be estimated as 5%, based on the mean value reported in previous works for A-frame systems.^11,30,31^ The power *W*_*Turb*_ is determined using the relation *PK*_3_, as seen in eq 11.

$$W_{net,t,l}=W_{Turb,t,l}\cdot \left(1-\frac{{Cons}_{DC}}{100}\cdot y_{DC,l}\right)\forall t\in TD,\forall l\in Loc$$ 10

$$W_{Turb,t,l}=PK_{3}\cdot m_{tank1-split1,t,l}\forall t\in TD,\forall l\in Loc$$ 11

The cooling requirements *Q*_*cool*_ (kW) and the heat flows of each heat exchanger (*Q*_*HX*1_, *Q*_*HX*2_, *Q*_*HX*3_, and *Q*_*HX*4_, kW) can be calculated by applying eqs 12–16:

$$Q_{cool,t,l}=m_{tank1-split1,t,l}\cdot PK_{4}\forall t\in TD,\forall l\in Loc$$ 12

$$Q_{HX1,t,l}=m_{tank1-split1,t,l}\cdot PK_{5}\forall t\in TD,\forall l\in Loc$$ 13

$$Q_{HX2,t,l}=m_{tank1-split1,t,l}\cdot PK_{6}\forall t\in TD,\forall l\in Loc$$ 14

$$Q_{HX3,t,l}=m_{tank1-split1,t,l}\cdot PK_{7}\forall t\in TD,\forall l\in Loc$$ 15

$$Q_{HX4,t,l}=m_{tank1-split1,t,l}\cdot PK_{8}\forall t\in TD,\forall l\in Loc$$ 16

Each cooling system has pros and cons for power or water consumption during operation. On the one hand, dry-cooling systems require an additional power consumption (*Cons*_*DC*_), but no water consumption is involved during the operation. On the other hand, wet-cooling technologies do not require additional power consumption, but there is a water consumption associated (*Wa*_*req*_, L_water_/kWh_prod_) to its operation due to the evaporative cooling. The relationship between the energy produced (kWh) by the facility and the water required was investigated in previous work for regenerative Rankine cycles, and it is presented in eq 17:^32^

$${Wa}_{req,t,l}=\left(\begin{array}{c} -2.297\cdot 10^{-4}\cdot T_{t,l}^{2}+0.798\cdot H_{t,l}^{2}\\ +7.090\cdot P_{t,l}^{2}+2.200\cdot 10^{-2}\cdot T_{t,l}H_{t,l}\\ +2.993\cdot 10^{-2}\cdot T_{t,l}P_{t,l}-0.515\cdot PH_{t,l}\\ -1.533\cdot 10^{-2}\cdot T_{t,l}-1.417\cdot H_{t,l}\\ -12.574\cdot P_{t,l}+7.6256 \end{array}\right)\forall t\in TD,\forall l\in Loc$$ 17

where *T* is the ambient temperature (°C), *H* is the relative humidity of air (kg/kg), and *P* is the pressure (bar), with each variable related to an *l* location and a *t* period. In the case of *y*_*WC*_ = 0, the variable *Wa*_*req*_ should be considered as 0.

### 2.3 Equipment Cost Estimation

Equipment costs were estimated per unit using piecewise linear approximations (see Supporting Information Section S2).

### 2.4 Social Impact of the Facility Location

Nowadays, one of the biggest concerns about the transition in the production system is the social impact related to it. In this work, the equation presented in previous works^33,34^ was adapted to include the corresponding annual salary per employee in each region (*Salary*, €_2025_/Job), as seen in Supporting Information Section S3. The social and salary data were obtained from public Spanish governmental databases.^35–37^

### 2.5 Environmental Impact of Facility Deployment

The environmental impact of the facility can be related to the use of a certain type of cooling system. In the case of DC systems, the environmental impact is associated with the power consumption by fans (*W*_*con*_, kW), which is calculated in eq 18 considering *y*_*DC*, *l*_ = 1:

$$W_{con,t,l}=W_{Turb,t,l}\cdot \frac{{Cons}_{DC}}{100}\forall t\in TD,\forall l\in Loc$$ 18

In the case of WC systems, the environmental impact related to the water consumption (*Wa*_*con*_, L/month) has a different concern according to the location because of the relative water availability. Thus, additional constraints and assessment methods are required to capture the effect of these variables in decision-making. The detailed model for water consumption impact is presented in Supporting Information Section S4.

## 3 Optimization Procedure

### 3.1 Problem Reformulation

Previous studies have addressed facility location and energy system optimization using mixed integer nonlinear programming (MINLP) formulations,^38^ which highlighted the computational challenges posed by bilinear terms and discrete decisions in large-scale problems. Building on these insights, the optimization problem is formulated as a mixed integer linear programming (MILP) problem, which preserves computational tractability and convergence reliability while providing sufficiently accurate results to support strategic CSP deployment and sustainability planning at the national scale, and the optimization was carried out using GAMS software.^39^ Most of the equations of the model are linear; however, constraint inequalities of design variables such as eq 12 show potential issues during the optimization because they can take large values: the larger the equipment, the larger the social impact. Thus, their formulation is extended by applying a BigM approach and introducing a new binary variable (*b*_*eq*,*t*,*l*_^*contr*^) and a positive intermediate design variable (*Var*_*eq*,*t*,*l*_^*contr*^) to restrict the feasible values: the positive values are calculated using *Var*_*eq*,*t*,*l*_, the BigM value *BM*_*eq*_, and *b*_*eq*,*t*,*l*_^*contr*^ for the selection of the design month; for each location *l*, only one *b*_*eq*,*t*,*l*_^*contr*^ can be selected; the corresponding design value for the variable *Var*_*eq*,*l*_^*des*^ is established with the month selected. Presented is the extended set of equations for the *Q*_*cool*_^*des*^ case in eqs 19–24:

$$Q_{cool,t,l}^{contr}\geq 0\forall t\in TD,\forall l\in Loc$$ 19

$$Q_{cool,t,l}^{contr}\geq Q_{cool,t,l}-{BM}_{cool}\cdot \left(1-b_{cool,t,l}^{contr}\right)\forall t\in TD,\forall l\in Loc$$ 20

$$Q_{cool,t,l}^{contr}\leq Q_{cool,t,l}\forall t\in TD,\forall l\in Loc$$ 21

$$Q_{cool,t,l}^{contr}\leq {BM}_{cool}\cdot b_{cool,t,l}^{contr}\forall t\in TD,\forall l\in Loc$$ 22

$$\sum_{t=1}^{TD}b_{cool,t,l}^{contr}=1\forall l\in Loc$$ 23

$$Q_{cool,l}^{des}=\sum_{t=1}^{TD}Q_{cool,t,l}^{contr}\forall l\in Loc$$ 24

This reformulation should be applied to the following variables: *Q*_*SR*_^*des*^, *Q*_*HX*1_^*des*^, *Q*_*HX*2_^*des*^, *Q*_*HX*3_^*des*^, *Q*_*HX*4_^*des*^, *W*_*Turb*_^*des*^. However, in the case of variables that are not design variables, or nonlinear equations (e.g., eq 10), the formulation should be expressed using the binary variables *y*_*ff*,*l*_, *y*_*DC*,*l*_, or *y*_*WC*,*l*_ instead of *b*_*eq*,*t*,*l*_^*contr*^ variables; this second formulation is mainly applied to variables and equations presented in the Supporting Information (see Section S2). An example of the formulation is given for the case of *Cost*_*WC*_^*des*^ in eqs 25–28. This reformulation should be applied to *W*_*net*_, *Cost*_*WC*_^*des*^, *Cost*_*DC*_^*des*^, *Cost*_*SR*_^*des*^, *Cost*_*tank*1_^*des*^, *Cost*_*tank*2_^*des*^, *Cost*_*Turb*_^*des*^, *Cost*_*HX*1_^*des*^, *Cost*_*HX*2_^*des*^, *Cost*_*HX*3_^*des*^, *Cost*_*HX*4_^*des*^, *Cost*_*ground*, *l*_, *Cost*_*hel*_, *W*_*con*_, and *Wa*_*con*_.

$${C\mathrm{os}t}_{WC,l}^{des}\geq 0\forall l\in Loc$$ 25

$${C\mathrm{os}t}_{WC,l}^{des}\geq {C\mathrm{os}t}_{WC,l}-{BM}_{C\mathrm{os}t,cool}\cdot \left(1-y_{WC,l}\right)\forall l\in Loc$$ 26

$${C\mathrm{os}t}_{WC,l}^{des}\leq {C\mathrm{os}t}_{WC,l}\forall l\in Loc$$ 27

$${C\mathrm{os}t}_{WC,l}^{des}\leq {BigM}_{C\mathrm{os}t,cool}\cdot y_{WC,l}\forall l\in Loc$$ 28

### 3.2 Objective Function

The objective function *Z* (€_2025_) includes the importance of the contributions considered in the model, as seen in eq 29: (1) cost of facilities, (2) environmental impact, (3) social impact, (4) production-related issues (power surplus or additional requirements), and (5) national taxes related to the social insurance cost (*NC*, €).^40^ The units of the contributions must be homogeneous to compare the importance of each term adequately, so contributions (2) and (3) were expressed in monetary units.

$$Z={PP}_{1}\cdot Z_{\cos t}+{PP}_{2}\cdot EI-{PP}_{3}\cdot SI+{PP}_{4}\cdot Z_{produc}+{PP}_{5}\cdot NC$$ 29

where *PP*_1_, *PP*_2_, *PP*_3_, *PP*_4_, and *PP*_5_ represent the priority parameters for investment, environmental impact, social impact, surplus, energy purchase, and taxes, respectively. In this work, it is assumed that *PP*_1_ = *PP*_2_ = *PP*_3_ = *PP*_4_ = *PP*_5_ = 1 because no additional specific priorities were considered.

The environmental impact was computed as the equivalent amount of CO_2_ emissions related to facility building and operation during the entire lifespan (35 years) (*CEI*_*fac*_, kgCO_2_eq). The Ecoinvent 3.10 database was employed considering the construction and maintenance CO_2_ emissions (*CEI*_*B*&*O*_, kgCO_2_eq) for a CSP tower plant of 20 MW (Rest of the World),^41^ applying the criteria from IPCC GWP 2011 and including CO_2_ uptake; the reference value was 5.43 × 10^7^ kgCO_2_eq/20MW for the entire lifespan of a CSP facility, without considering a cooling system. The estimation of *CEI*_*fac*_ is shown in eq 30:

$${CEI}_{fac}={CEI}_{B\&O}\cdot \sum_{l=1}^{Loc}W_{Turb,l}^{des}\cdot \frac{1}{{LE}_{fac}}$$ 30

The water consumption (*Wa*_*con*_) or the power consumption (*W*_*con*_) depend on the cooling system, as seen in eqs 31 and 32, where *CEI*_*water*_ is the environmental CO_2_ contribution related to water consumption (kgCO_2_eq), *CEI*_*power*_ is the environmental CO_2_ contribution related to power consumption (kgCO_2_eq), *R*_*L*→*CO*2_ is the ratio of kgCO_2_eq/m^3^H_2_O (0.30 kgCO_2_eq/m^3^H_2_O),^11^ and *R*_*kWh*→*CO*2_ is the ratio of kgCO_2_eq/kWh consumed (0.322 kgCO_2_eq/kWh):^42^

$${CEI}_{water,t,l}={WX}_{l}\cdot {Wa}_{con,t,l}\cdot R_{L\to CO2}\forall t\in TD,\forall l\in Loc$$ 31

$${CEI}_{power,t,l}=W_{con,t,l}\cdot {days}_{t}\cdot OP\cdot R_{kWh\to CO2}\forall t\in TD,\forall l\in Loc$$ 32

Thus, the total environmental impact for the system (*EI*, €_2025_) is calculated as seen in eq 33, where *R*_*CO*2→*$*_ is the price of kgCO_2_eq (0.15 €_2025_/kgCO_2_eq):^43^

$$EI=\left[{CEI}_{B\&M}+\sum_{t=1}^{TD}\sum_{l=1}^{Loc}\left({CEI}_{water,t,l}+{CEI}_{power,t,l}\right)\right]\cdot R_{CO2\to \$}$$ 33

The economic impact of the power surplus (*Surp*_*power*_, €) is defined by eq 34 in the case of *W*_*add*,*t*_ = 0 and *Surp*_*power*_ = 0 in the case of *W*_*add*,*t*_ > 0; meanwhile, the economic impact of an additional power requirement (*Diff*_*power*_, €_2025_), eq 35, is employed to compute *W*_*add*_ contribution considering a penalty cost *Penalty*_*kWh*→*$*_ (assumed 5.00 €_2025_/kWh).

$${Surp}_{power}=\overset{TD}{\underset{t=1}{\sum }}\left[\overset{Loc}{\underset{l=1}{\sum }}\left(W_{net,t,l}\right)-W_{Dem,t}\right]\cdot R_{kWh\to \$}$$ 34

$${Diff}_{power}=\sum_{t=1}^{TD}\left(W_{add,t}\right)\cdot {Penalty}_{kWh\to \$}$$ 35

Thus, the contribution of the production of power to the objective function (*Z*_*produc*_, €) is shown in eq 36, and the cost contribution (*Z*_*cost*_, €) is shown in eq 37:

$$Z_{produc}={Diff}_{power}-{Surp}_{power}$$ 36

$$Z_{\cos t}=\sum_{l=1}^{Loc}\left({\cos t}_{fac,l}\right)\cdot \frac{1}{{LE}_{fac}}$$ 37

The optimization problem to solve is defined as seen in eq 38:

minZs.t.eqs.(1)−(37),(S37)−(S103) 38

## 4 Case Study: Spain

In this work, Spain is selected as a case study to evaluate the model because it is a country with a large solar irradiance and a promising solar technology development perspective.^44^ However, the archipelagos and the African territories of Spain are not considered because they are isolated from the national grid or do not present a suitable place to build a solar plant. Spanish data are collected in the Supporting Information (Section S5). The Spanish territory is divided into 47 provinces (see Figures S2 and S3A) considering the official Spanish NUTS-3 distribution; in this regard, *DNI*, water availability, and social data have been defined with the same discretization scale because of three reasons: (1) each region covers a relatively small area in terms of employee relocation and therefore does not pose a significant barrier to intraregional mobility; (2) NUTS-3 resolution is enough for conceptual studies regarding *DNI*, water, and social indices values; and (3) high-tension lines are available in every region,^45^ and there are several grid extension projects in progress.^46^ The time frame considered for the data is one year with a monthly discretization step. Social impact data are computed as social ratios, which are the quotients in parentheses of eqs. S96–S98; the social ratios per Spanish province show an uneven distribution throughout the territory, with most of the population in large cities, such as Madrid (region #20) (see Figure S3B). In the case of GDP, the highest values are presented in the northeastern region of the country and the capital (see Figure S3C). These data can partially explain the exodus from towns to cities, looking for better profit and employment opportunities (Figure S3D).

## 5 Results and Discussion

### 5.1 Evaluation of Model Performance

The detailed plant model presented in the Supporting Information (see Section S1) was evaluated and validated in previous works.^25,26^ Regarding the location selection problem, a small study with three locations and five scenarios was proposed to evaluate the robustness of the optimization problem (see Section S6). The solutions show consistent results for each scenario because most selected locations toward the south of the country also meet the criteria.

### 5.2 Evaluation of Locations Considering All Provinces

The whole country facility location problem was evaluated by using progressive demand substitution scenarios. These scenarios were defined based on the average share of national electricity demand supplied by each nonrenewable source and nuclear power.^47^ Table 2 presents five substitution scenarios (A–E), ranging from coal-based generation to a fully renewable system.

**Table 2:** Substitution of Energy Sources per Scenario^a^

**scenario**	**energy sources considered**	**average relative amount of national demand (%)**
A	C	1.92
B	C + Di + TG + RNR	3.91
C	C + Di + TG + RNR + CC	21.03
D	C + Di + TG + RNR + CC + COG	31.06
E	C + Di + TG + RNR + CC + COG + NuR	51.86

a C: Coal; Di: diesel engines; TG: gas turbines; RNR: nonrenewable wastes; CC: combined cycle; COG: cogeneration; NuR: nuclear reactors.

As a general result, dry-cooling technology is selected in every scenario because additional power consumption leads to an increase in social impact and larger investment: the lower the net energy production, the larger the turbine installed and the larger the number of jobs created, as seen in eqs S96–S98. This finding underscores a critical yet underexplored trade-off between energy efficiency, investment costs, and social benefits, offering a novel perspective on solar energy deployment strategies. Beyond current conditions, future climate projections for Spain indicate increasing water scarcity and stricter constraints on cooling water use in the coming decades, as reported by national water scarcity and drought assessments.^48^ Consequently, the preference for dry-cooling systems identified in this work is expected to be reinforced under future climate and regulatory conditions rather than reversed.

Figure 2 depicts the distribution of the production capacity for each scenario. It can be noted that the regions in the south of Spain are progressively selected to meet the demand, beginning with region #1 (Almeria) and gradually expanding across the southern part of the country. According to the monthly *DNI* data (see Figure S5), while the solar resources in southern Spain are lower than in central regions during the summer, they exhibit higher and more stable values throughout the rest of the year. This stability could facilitate the sizing of the facilities by mitigating fluctuations.

**Figure 2:**
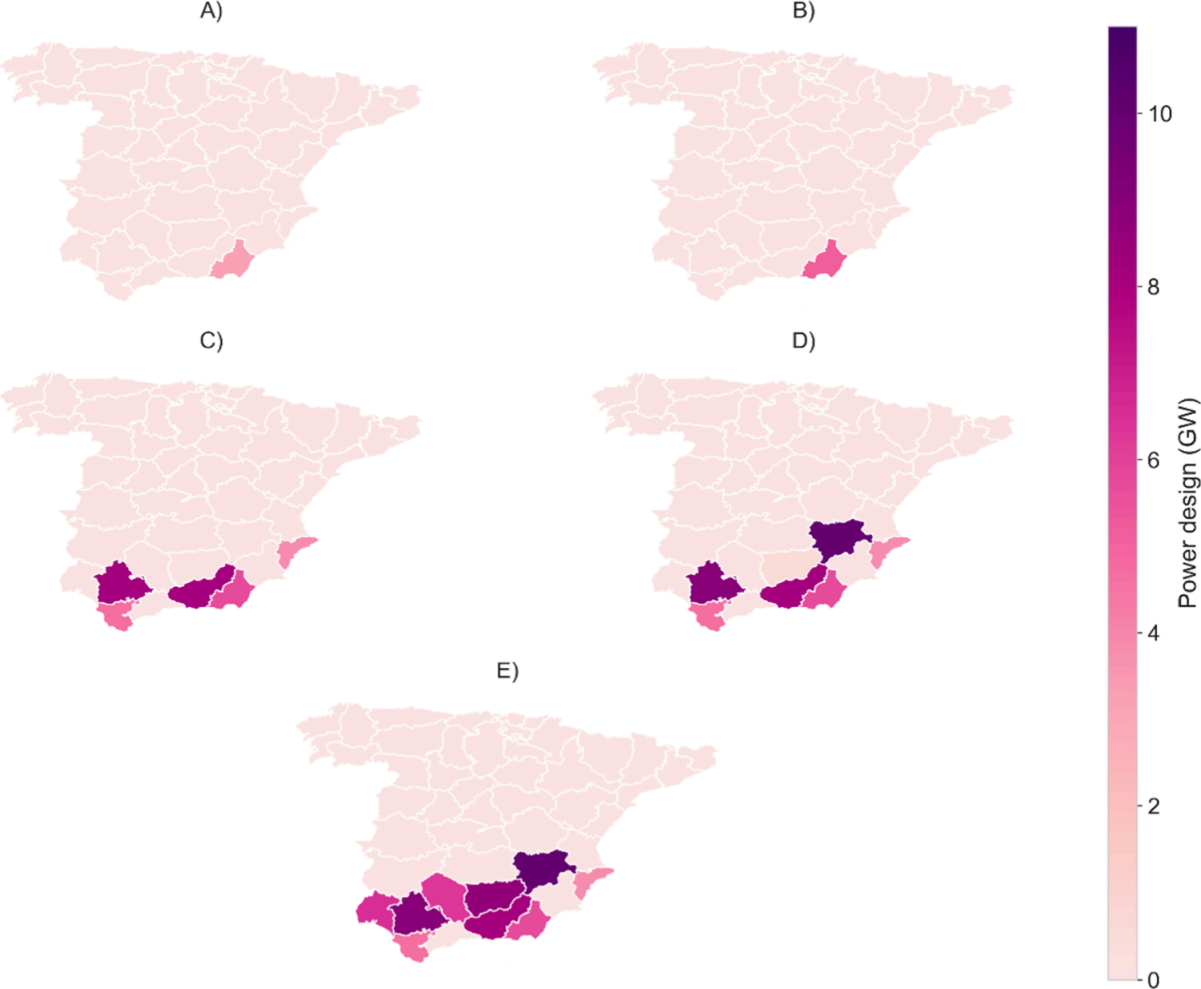
Distribution of CSP production capacity across Spain for each scenario, showing the progressive selection of southern regions, based on *DNI* and available land, to meet increasing demand while balancing solar resource availability and land constraints.

As shown in Figure 3, once the maximum allowable area in a given region is occupied (0.5%), additional regions are selected to meet the energy demand. This highlights one of the key limitations of solar energy: the larger the energy demand, the larger the area required. Furthermore, the results remain consistent, as the total available area of previously selected regions is fully occupied before a new one is chosen, considering any scenario. Nevertheless, in Scenario D, instead of selecting regions from Andalucia, the model chose region #12 (Albacete). This selection may be related to the social development benefits expected by the facility deployment, which are much larger in region #12 than in the previous scenarios (A and B) and subsequent ones (D and E), as shown in Figure 4.

**Figure 3:**
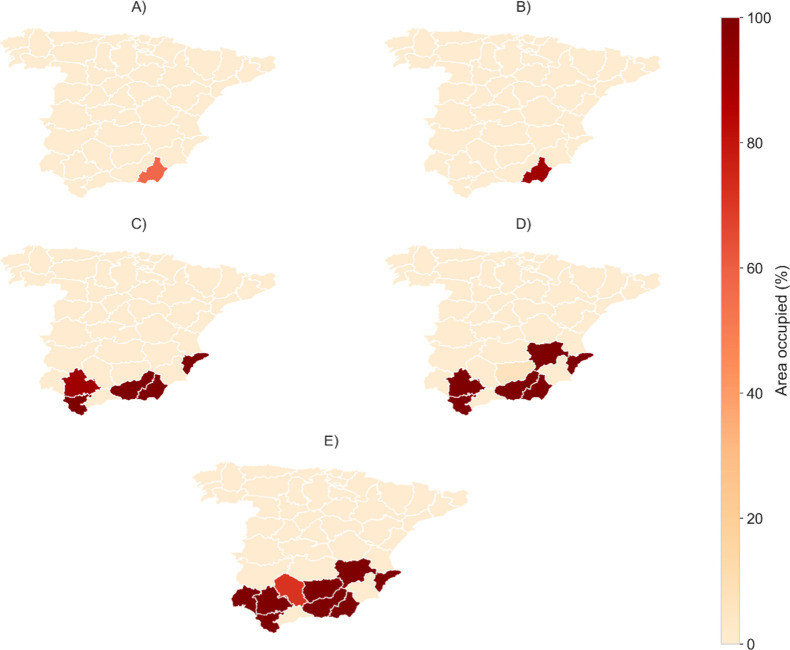
Percentage of the maximum allowable area occupied in each region for each scenario. Additional regions are selected once the area limit in a region is reached.

**Figure 4:**
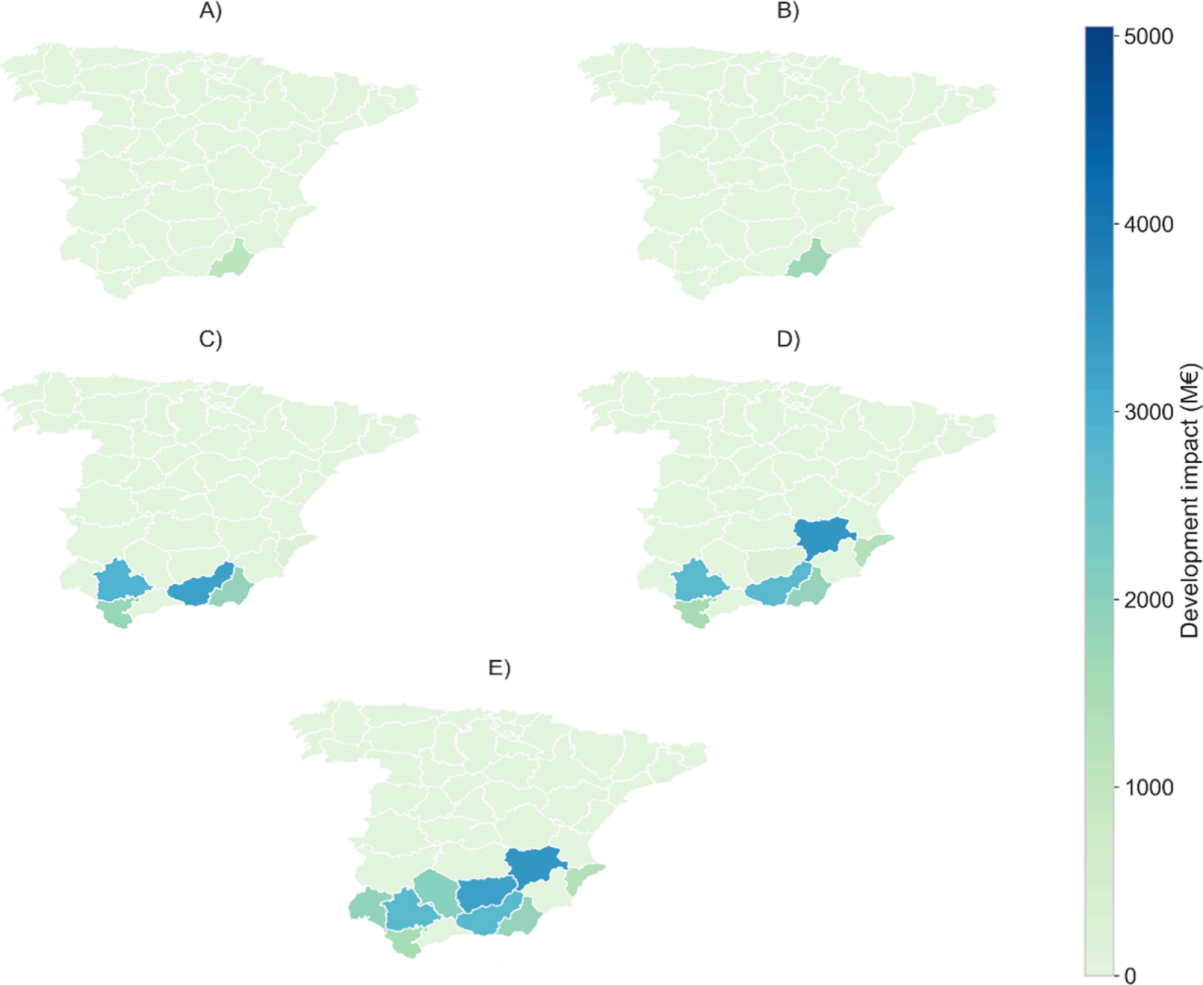
Estimated social development impact for each scenario, including job creation and regional benefits. Regions are selected due to higher social development potential compared to others with slightly higher *DNI* or lower costs.

This result challenges conventional economic preferences, such as minimizing investment costs while producing the same amount of energy or reducing investment costs relative to subsequent scenario choices, as shown in Figure 5. The consideration of social impact affects the decision-making process in renewable energy deployment, establishing a novel trade-off between monthly radiation, investment costs, and social impact, rather than optimizing one of them.

**Figure 5:**
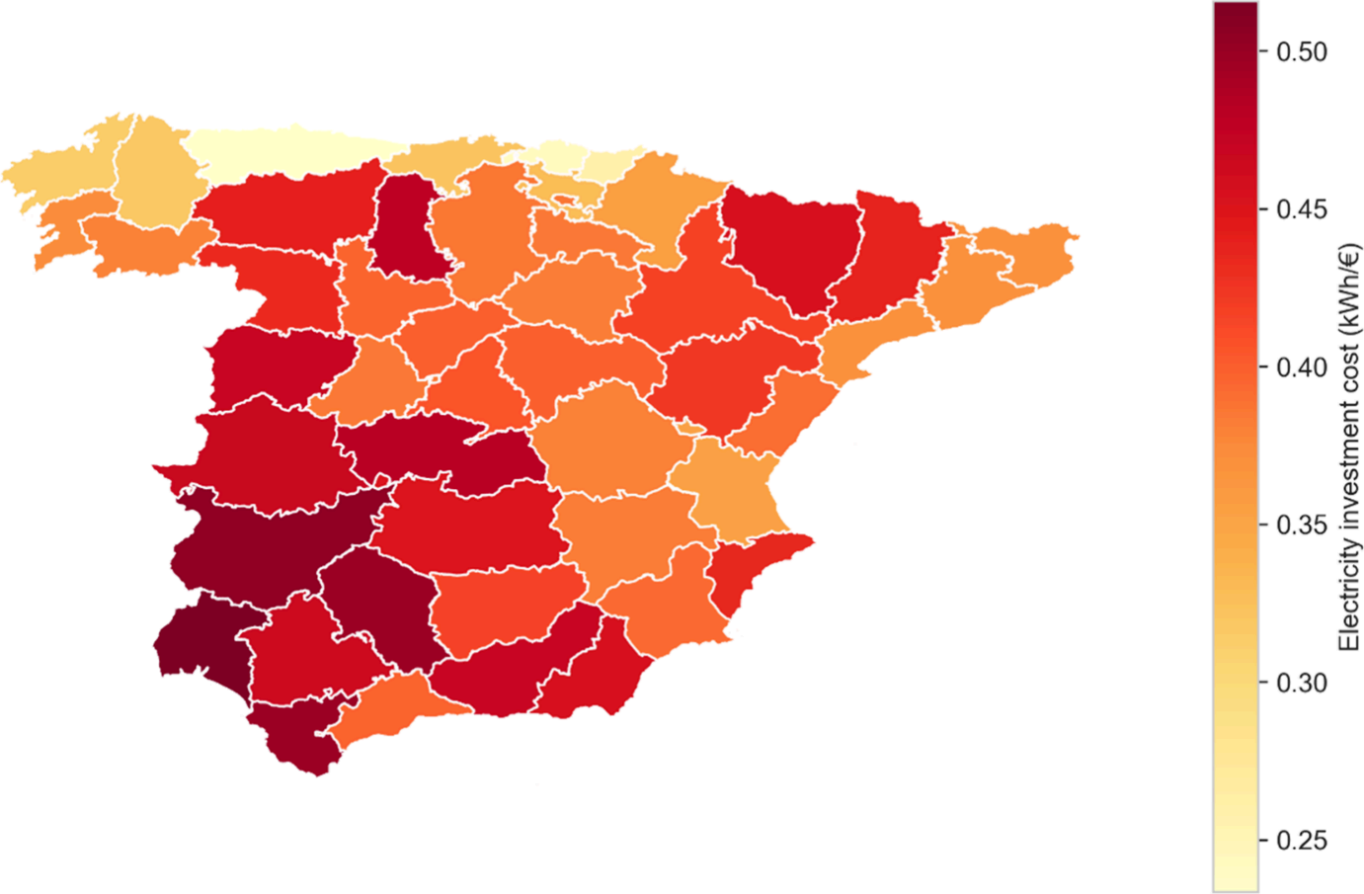
Investment cost per kWh for each region. The figure illustrates the variation in economic investment across regions for CSP deployment.

Originally, the selection of regions was decided according to the values of *DNI* for December (see Figure S5) because it is the most challenging month for meeting the energy demand by using solar radiation. As a priority, the region with the largest *DNI* (region #1, Almeria) is chosen in scenarios A and B, until the maximum area allowed is occupied. After that, other regions with high *DNI* values are selected in scenario C (regions #4 [Granada] and 17 [Alicante]). Regions #2 (Cadiz) and 8 (Sevilla) are also selected among the other regions with similar *DNI* values and lower investment (see Figure S6). These regions are chosen based on the ratio kWh/€ (Figure 5) or higher social impact values (Figure 4), as region #2 presents a better economic ratio, while region #8 shows notably better social development potential. This result highlights that when the investment costs are similar, the importance of other factors such as the social impact associated with the investment plays a crucial role in decision-making.

Regarding social impact, region #12 (Albacete) presents higher values than the other southern regions, mainly due to job creation and depopulation reduction. Nevertheless, comparing Figure 5 and the maximum achievable social impact (see Figure S7), it would be expected that the next region chosen would be #11 (Badajoz) when moving to scenarios C to D, given its lower cost and substantial social impact. However, Figure 4 shows that the model selects region #12 instead. This can be explained by the objective function, eqs 29 and 38, as follows: (1) Region #11 presents higher *DNI* values throughout the year and a larger available area, implying greater attainable production capacity (see Figure S8), but this also increases the value of *Surp*_*power*_ considering the joint operation of all facilities, which is penalized according to eq 34; (2) a larger production capacity leads to higher investment (see Figure S9), which is a variable to minimize as seen in eq 37, even though an increased capacity might provide some positive benefits due to larger *W*_*Turb*_^*des*^ (eqs S96–S98); and (3) the environmental impact in region #11 could be higher than in region #12 due to the relationship between the energy production every month and the energy consumption by dry-cooling technology, eq 10: the greater the production, the higher the consumption.

In scenario E, the regions chosen are located in the South (#3, 5, and 6) again due to the better trade-off between the available radiation and social impact. Region #6 is selected first due to its higher social development potential. It is important to note that those regions were chosen instead of #10 or 11 due to their higher *DNI* values for December.

From the analysis presented above, a structured decision-making procedure can be inferred for regional selection in solar energy deployment, prioritizing the following: (1) regions with the highest *DNI* in December are preferred, as this is the most challenging month for energy production; (2) when DNI differences are not significant, the cheapest option is selected; and (3) if investment and *DNI* values are similar, the region with higher social impact potential is prioritized. These results highlight that explicit inclusion of social indicators such as unemployment, regional GDP, or population density shifts the optimal allocation toward southern and inland regions with greater socioeconomic needs, in line with previous renewable energy planning studies in Spain conducted at comparable system scales.^33,49^

In order to evaluate the sensitivity of the spatial allocation to land availability assumptions, an additional sensitivity analysis of the maximum allowable ground footprint per province was conducted. The analysis considered allowable footprints of 0.25, 0.50, 0.75, and 1.00% of the provincial area (see Section S8 in the Supporting Information). Results show that lower allowable footprints (0.25%) require CSP plants to be distributed across up to 8 additional provinces to meet the target capacity, which increases overall investment costs by up to 14%. In contrast, higher allowable footprints (0.75–1.00%) lead to more concentrated deployments, with provinces exhibiting the highest *DNI* remaining preferentially utilized, while installed capacity in secondary regions is progressively reduced, resulting in a cost reduction of up to 122 B€ for the 1.00% allowable footprints.

### 5.3 Economic Estimations and Social-Environmental Impacts

The economic evaluation of the conceptual CSP network is presented in Table 3, considering a discount rate of 7%^50^ and 120 €/kWh^51^ for NPV calculations. The green transition to a fully decarbonized electricity system is achievable using CSP technology, with a budget target of approximately 785 B€_2025_. These results, which align with previous sustainable studies,^52^ show that a substantial effort from governments and the industrial sector would be required to tackle funding issues, for example, with subsidies. Even though the levelized cost of electricity (LCOE) remains within a competitive range (0.086–0.093 €/kWh), NPV results discourage the investment because they are negative in any scenario. A possible approach to deal with economic issues could be negotiating a fixed electricity sell price for the CSP plants built to substitute fossil fuels around 136 €/MWh and considering a return ratio of 3% instead of 7% (see Supporting Information, Section S9).

**Table 3:** Economic Metrics per Scenario

**item**	scenario 1	scenario 2	scenario 3	scenario 4	scenario 5
CSP capacity (GW)	3.25	5.16	30.43	41.83	62.73
net energy produced (GWh)	17,723	29,700	171,724	233,811	349,162
**CAPEX**					
CSP investment (M€_2025_)	42,452	67,041	375,979	534,209	785,220
**OPEX**					
O&M (M€_2025_)	8,490	13,408	75,196	106,842	157,044
insurance and taxes (M€_2025_)	637	1,006	5,640	8,013	11,778
miscellaneous (M€_2025_)	425	670	3,760	5,342	7,852
**Economic Metrics**					
LCOE (€/kWh)	0.091	0.090	0.086	0.093	0.090
NPV (M€) (35 years, 7%, 120€/MWh)	–20,107	–31,414	–165,549	–258,917	–367,171

Nevertheless, in recent years, several global and political events have produced significant electricity price fluctuations,^53^ such as the COVID-19 pandemic (2020) and the war in Ukraine (2022). The evolution of LCOE as a function of discount rate (Figure 6) illustrates the subsidies required to achieve an NPV of zero under different rates. At current prices (2025), a maximum discount rate of 2% can be affordable; by contrast, 2022 prices will produce benefits for the same rate. However, prices like those of 2020 would require subsidies of approximately 40 €/MWh to avoid economic losses. The required subsidy, expressed in €/kWh, falls within a relatively narrow interval and remains nearly constant across the different scenarios (Figures S19–S23). Scenario C presents slightly lower values, which can be attributed to numerical accuracy and differences in land occupation costs among regions. The complete evaluation of the required subsidy for each scenario, considering different electricity market prices and discount rates, is provided in the Supporting Information (see Section S10). These results highlight the importance of establishing a minimum electricity price as a mechanism to support the energy transition.

**Figure 6:**
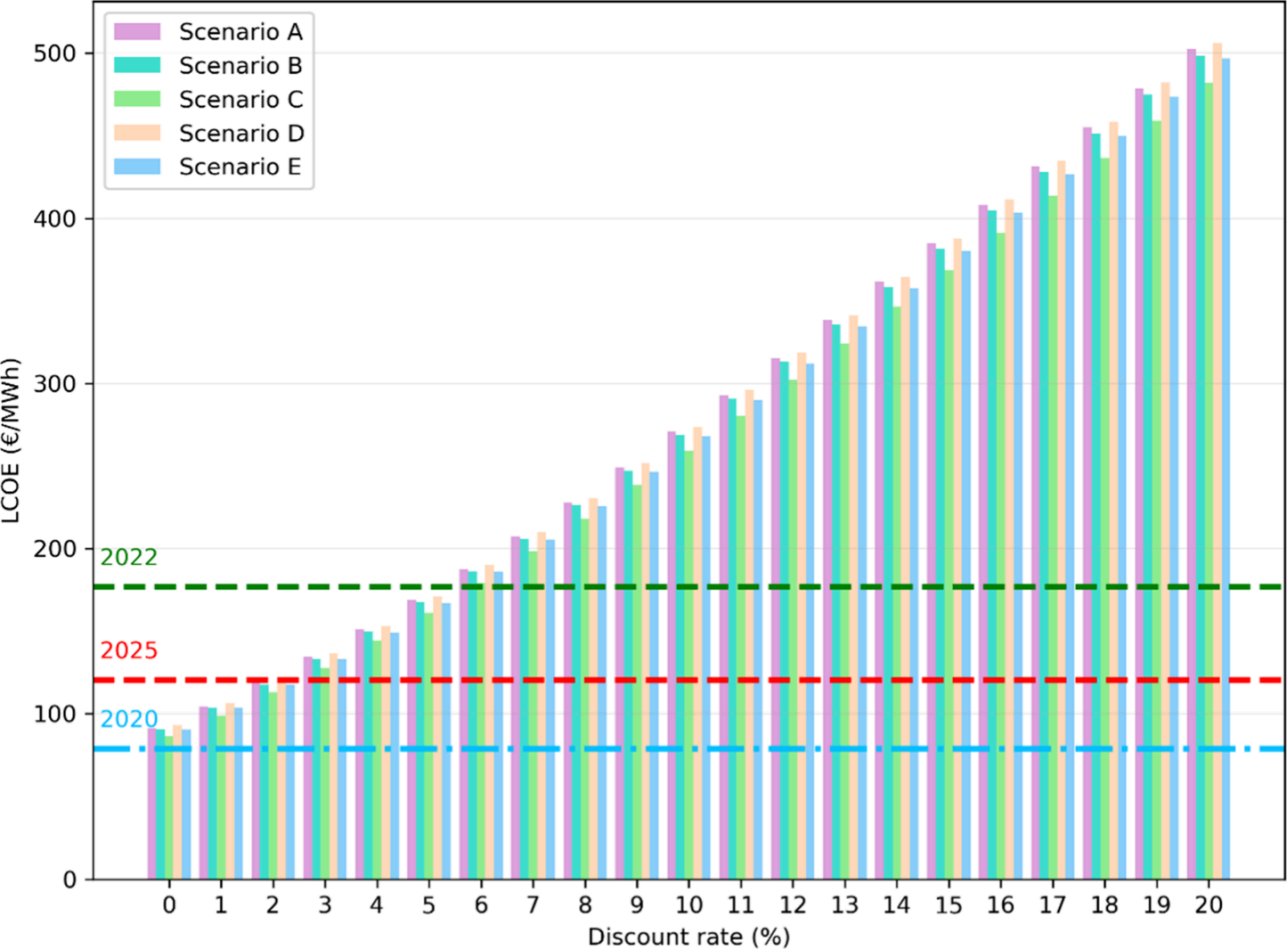
Effect of discount rate on LCOE for each scenario, compared with historical Spanish electricity values for 2020, 2022, and 2025. This comparison highlights how CSP profitability and the required policy support vary with market conditions.

The social and environmental impacts of each scenario are presented in Table 4. Indirect jobs created are estimated to be 7.5 times the direct jobs.^54^ The number of jobs created increased with investment, and the selection of dry cooling technologies instead of wet cooling leads to the creation of a larger number of jobs. This is explained by a trade-off between dry cooling and power capacity: the larger the dry cooling system, the larger the power capacity of the plant and, thus, the larger the number of jobs created. Compared to current data,^35^ these results suggest that the deployment of CSP plants can potentially reduce national unemployment by 2.5% (direct jobs) and 21% (total jobs), at 100% renewable power production. These values should therefore be interpreted as a maximum potential impact rather than a conservative projection and are consistent in order of magnitude with institutional assessments of employment impacts from large-scale renewable energy deployment in Spain and internationally.^55,56^ Regarding CO_2_ emissions, reductions in any scenario are notably positive, as avoiding the use of cooling water and fossil fuels prevents the generation of additional emissions, even though the total cumulative power capacity is required to be slightly higher.

**Table 4:** Environmental and Social Impacts per Scenario

**scenario**	**investment budget (M€**_**2025**_ **)**	**direct jobs created**	**indirect jobs created**	**CO**_**2,Tech**_ **– CO**_**2,CSP**_ **(ton CO**_**2**_ **eq)**
1	42,452	3,255	24,412	370,983
2	67,041	5,162	38,715	1,039,593
3	375,979	23,049	172,868	5,103,489
4	534,209	49,759	373,192	8,157,753
5	785,220	62,731	470,482	14,991,235

To further analyze the relationship between economic effort and social impact, a Pareto-type analysis was carried out by grouping all cost-related terms into a single economic objective and evaluating them jointly against the social impact term (Supporting Information Section S11). Moderate increases in investment are associated with noticeable improvements in social impact at low impact levels, while further gains require increasingly higher investment, approaching an approximately linear trend. At high social weights, the model tends toward the imposed maximum investment limit, which is consistent with an optimization predominantly driven by social objectives.

The relationship between power design and investment cost across different regions was analyzed, considering the maximum area occupied, to develop a conceptual equation for estimating the total equipment investment cost, given by eq 39 (Figure S25, Supporting Information Section S12). The estimation fits the data acceptably or falls within a 95% confidence interval. The fluctuations in costs are related to the different regional factors, such as achievable power capacity, land price, etc. Nevertheless, there is an outlier, which corresponds to region #41; this is explained by its more expensive cost per kWh, as seen in Figure 5, which justifies the pronounced vertical displacement (see Figure S25). This highlights the influence of regional economic conditions on investment costs and remarks on the need for location-specific financial considerations in energy planning.

$${Invest}_{fac}=0.01\cdot W_{Turb}^{des}+15.27$$ 39

For design purposes, a different design month is identified depending on the facility region, as shown in Figure 7. In addition, it is possible to identify two design groups: (1) equipment related to the heliostat field (solar receiver and heliostats) and (2) other equipment comprising the thermal cycle. On the one hand, it can be noted that different months are selected for heliostat field-related equipment in some regions, regardless of whether they are located in the same part of the country. This is due to the *DNI*/*H*_*sun*_ ratio of each region (see Figure S10): the larger the ratio, the more demanding the solar receiver operation. On the other hand, the thermal cycle equipment is designed considering June or July, corresponding to the month of highest *DNI* values for each region. These results demonstrate that the most demanding operation for the solar receiver and turbine system is not necessarily aligned due to the effect of thermal storage suppressing the daily variability of solar radiation: even though the values of *DNI* are larger during summer, the proportional increment of *H*_*sun*_ is more significant. Thus, the heat flow through the solar receiver is reduced compared to other months, such as April. However, the turbine system work is increased due to the higher number of working hours of the solar receiver, and so the water steam mass flow is used to produce electricity. Only regions #44 (Gipuzkoa) and 45 (Bizkaia) present the same month for both designs, which can be explained by the fact that there are the regions with the lowest *DNI* and *H*_*sun*_ for each month. These results align with previous works.^57^

**Figure 7:**
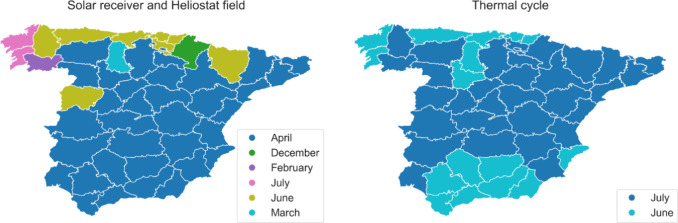
Design month for the heliostat field (solar receiver and heliostats) and thermal cycle equipment (rest of the facility) in each region. Regional solar conditions influence the timing of facility design for optimal operation, and electricity demand affects installed power capacity.

### 5.4 Insights and Recommendations

The results from Sections 5.2 and 5.3 provide practical guidance for national-scale CSP deployment: (i) Dry-cooling is selected across all scenarios, reflecting a balance between net energy production, investment, and social benefits, which suggests that it should be considered as the standard approach for new plants. (ii) Land availability and *DNI* values drive regional selection, with phased deployment in southern regions allowing for efficient use of resources, stable energy production throughout the year, and enhanced social impact. (iii) The economic analysis indicates that CSP investments require supportive policies, such as fixed electricity prices or moderate subsidies, to become financially viable. At the same time, a large-scale deployment can generate substantial employment and reduce CO_2_ emissions, highlighting clear social and environmental benefits. (iv) Variations in the optimal design month for heliostat fields and thermal cycle equipment across regions further emphasize the need to tailor facility design to local solar conditions. Overall, these findings offer actionable insights for planners, policymakers, and investors, demonstrating how technical, economic, and social-environmental considerations can be integrated to support a gradual and sustainable expansion of CSP.

## 6 Conclusions

The design of the new energy system presents a challenge for the ongoing green transition of national electrical energy production systems. This work presents an optimization formulation to study the facility location of CSP plants across the country. The model includes the effect of location-related parameters (such as direct normal irradiance, temperature, pressure, and water availability) and the choice of cooling technologies per facility. It also incorporates social concerns and environmental impacts as decision-making criteria. The case study is Spain, a country with plenty of solar radiation and various climate zones. The evaluation was conducted by formulating a multiobjective mixed-integer linear programming (MILP) optimization model, tested with a smaller case study for robustness analysis.

The results showed that the regions located in the southern area were selected first to meet the energy demand during the winter. The subsequent choice of location depends on a trade-off between cost and social concerns while also considering the availability and variability of direct normal irradiance in the region. Furthermore, the selection of the design month for the equipment was characterized, and how to select it was defined: heliostat field-related equipment optimized for months with the highest *DNI*/*H*_*sun*_ ratio, while thermal cycle equipment was designed based on peak *DNI* months, typically June or July. A correlation for the regional equipment investment estimation as a function of production capacity was provided. The results also showed that substituting fossil fuel contributions with renewable energies is financially viable in the long term, with an estimated cost of 785 B€_2025_ and LCOE of approximately 0.086–0.093 €/kWh.

By incorporating socio-economic variables into the decision-making process, this study advances the understanding of how large-scale solar energy projects can be optimized for both efficiency and broader societal benefits. Unlike previous studies that primarily focus on maximizing *DNI* availability or minimizing costs, this work introduces a multicriteria location selection process, integrating economic and social dimensions alongside energy efficiency. Additionally, it highlights the importance of regional economic conditions, investment requirements, and job creation potential, demonstrating that CSP deployment could reduce national unemployment by approximately 2.5–21%, considering both direct and indirect jobs.

## Supplementary Material



## Notes

Detailed CSP plant model (eqs S1–S36) (Figure S1); cost estimation equations of equipment (eqs S37–S94) (Table S1); social impact of the facility location (eqs S95–S98); environmental impact of water consumption (eqs S99–S103); location related data (Figures S2–S5) (Tables S2 and S3); evaluation of model performance (eqs S104–S106) (Tables S4–S6); additional results of each case study (Figures S6–S10) (Table S7); sensitivity analysis of ground availability (Figures S11–S16); evaluation of NPV for each case study (Figures S17 and S18); evaluation of required subsidy (Figures S19–23); Pareto front for social impact and investment cost (eq S107) (Figure S24); investment cost per capacity installed (Figure S25) (PDF)

## NOMENCLATURE

**Sets**
Loc: Locations considered, 47 counties.
TD: Points of time discretization, 12 months.
**Abbreviations**
C: Coal.
CC: Combined cycle.
COG: Cogeneration.
CSP: Concentrated Solar Power.
DC: Dry cooling.
Di: Diesel engines.
DR: Development ratio.
GDP: Gross development per habitant.
HTF: Heat transfer fluid.
NuR: Nuclear reactors.
RNR: Nonrenewable wastes.
RP: Relative population.
TG: Gas turbines.
UR: Unemployment ratio.
WC: Wet cooling.
**Variables**
A: Area (m^2^).
b: Binary variable (−).
*C*_*p*_: Thermal capacity (kJ/kg K).
CEI: Contribution for environmental impact (kgCO_2_eq).
Cost: Cost of an equipment (€).
*Diff*_*power*_: Additional power required (kW).
DNI: Direct normal irradiance (kWh/(m^2^·d)).
EI: Environmental impact (€).
H: Relative humidity of air (kg/kg).
*H*_*sun*_: Hours of sun (h).
Invest: Investment (€).
m: Mass flow rate (kg/s).
n: Number of elements (−).
NC: National Insurance Cost (€).
P: Pressure (bar).
PK: Proportional constant (units in Table 1).
Q: Heat flow of a unit (kW).
SI: Social impact (€).
Social: Social index (€).
*Surp*_*power*_: Power surplus (kW).
T: Temperature (°C).
W: Power of a unit (kW).
*Wa*_*con*_: Water consumption (L/month).
*Wa*_*req*_: Water requirement (L/kWh).
WX: Water index.
y: Binary variable (−).
Z: Objective function (€).
*Z*_*cost*_: Costs term of the objective function (€).
*Z*_*produc*_: Production-related issues term of the objective function (€).
**Parameters**
*A*_*hel*_: Area of a heliostat (120 m^2^).
*BigM*: BigM value (related to each equation, above 100 times bigger).
*CEI*_*B* &*O*_: Construction and maintenance CO_2_ emissions (5.43·10^7^ kgCO_2_eq/20MW).
*Cons*_*DC*_: Power consumption by dry-cooling system (5%).
*F*: Decision weights for social indices.
*FWA*: Fraction of water available (10%).
*GDP*: Gross development per habitant (€/hab).
*Ground*_*ava*_: Ground availability for construction (0.5%).
*J*_*Solar*_: Jobs generated by the creation of the facility (1 Job/MW).
*LE*_*fac*_: Facility life span (35 years).
*n*_*fac*_^*max*^: Maximum number of facilities (47).
*OP*: Operation time (24 h).
*Penalty*_*kWh* → *$*_: Penalty cost (5.00 €_2025_/kWh).
*Population*: Population in the region.
*PP*: Priority parameter of objective function.
*R*_*kWh* → *CO*_2__: Ratio of kgCO_2_eq/kWh (0.322 kgCO_2_eq/kWh).
*R*_*L* → *CO*_2__: Ratio of kgCO_2_eq/m^3^H_2_O (0.30 kgCO_2_eq/m^3^).
*R*_*CO*_2_ → *$*_: Price of CO_2_ emissions (0.15 €_2025_/ kgCO_2_eq).
ρ_*inhab*_: Population density (hab/km^2^).
*Salary*: Annual salary per employee (€/Job).
*UV*: Unemployment value (hab).
**Greek letters**
η: Efficiency.
**Sub-indices and super-indices**
l: *l*th of set *Loc*.
t: *t*th of set *TD*.
Rad: Solar radiation.
*tank*1-*split*1: from Storage tank 1 to Splitter1.
SR: Solar receiver.
*tank*2-*SR*: from Storage tank 2 to Solar Receiver.
Turb: Turbines.
Cool: Cooling.
*HX*1: Heat Exchanger 1.
*HX*2: Heat Exchanger 2.
*HX*3: Heat Exchanger 3.
*HX*4: Heat Exchanger 4.
hel: Heliostat.
layout: Layout of the heliostat field.
Prov: Province.
Dem: Demand.
add: Additional.
net: Net production.
fac: Facilities
ff: Facility feasibility.
dc: Dry-cooling technology.
wc: Wet-cooling technology.
des: Design.
con: Consumption.
contr: Contribution.
produc: Production-related contributions.
