# Supplementary material for: Toward a Sustainable Energy Production System Based on Concentrated Solar Power Plants: Social and Water Availability Issues
Source: ACS Sustain Chem Eng. 2026 Feb 12;14(7):3431–45. doi: 10.1021/acssuschemeng.5c11213 (PMC12934526; doi:10.1021/acssuschemeng.5c11213)
Supplement: Supplementary file 1 [file sc5c11213_si_001.pdf]

SUPPORTING INFORMATION

TOWARDS A SUSTAINABLE ENERGY PRODUCTION SYSTEM BASED ON CSP PLANTS: SOCIAL AND WATER AVAILABILITY ISSUES

Jose A. Luceño-Sanchez †,‡*, Mariano Martin‡, Sandro Macchietto†

†Department of Chemical Engineering, Imperial College London, South Kensington Campus, London, SW7 2AZ, United Kingdom

‡ Department of Chemical Engineering, University of Salamanca, Plaza de los Caídos 1-5, Salamanca, 37008, Spain

Number of Pages: 42

Number of equations: 107

Number of Tables: 7

Number of Figures: 25

* Corresponding author. J.A. Luceño-Sánchez. jalucenyos@usal.es, j.luceno-sanchez@imperial.ac.uk

Table of Contents:

Page

S1. Detailed CSP plant model ---------- S-3

S2. Cost estimation equations of equipment ---------- S-9

S3. Social impact of the facility location ---------- S-14

S4. Environmental impact of water consumption ---------- S-15

S5. Location related data ---------- S-16

S6 Evaluation of model performance ---------- S-21

S7. Additional results ---------- S-23

S8. Sensitivity analysis of ground availability ---------- S-27

S9. Evaluation of NVP ---------- S-33

S10. Evaluation of required subsidy ---------- S-34

S11. Parefo front for social impact and investment cost ---------- S-39

S12. Investment cost per capacity installed ---------- S-40

S13. References ---------- S-40

S1. Detailed CSP plant model

Modeling assumptions

The flowsheet of the plant consists of three parts, the heliostat field including the collector and the storage tanks, the turbine system that uses the energy obtained from the sun to generate high pressure steam and from it, electricity, and the cooling tower that is used to cool down the low-pressure discharge [1]. Figure S1 presents the flowsheet for the process where the heliostat field has not been included. In this section we present the model of the units involved in the processes following the same path of the energy transferred, the collector, the turbine system and finally the cooling tower. Our process flowsheet is based on the use of a tower to collect the solar energy.

Collector and heliostats field

The solar energy is redirected to a tower using heliostats where the molten salts are used as heat transfer fluid (60%w/w $NaNO_3$–40% w/w KNO_3) [2]. Typically, heliostats size range from 70 to 150 m^2. In our case we consider a mean value, 120m^2. The tower has a heat exchanger in the top that is used to heat up the molten salts by the concentrated solar energy. Calculating the efficiency of a heliostat field requires evaluating the most significant factors influencing the performance of the heliostat field: cosine losses (20%), shading and blocking (2% losses) [3], heliostat reflectivity (typical efficiency values range from 0.90 to 0.95 [4] and transmission losses through the atmosphere (5% losses) [3]. To be on the safe side we consider a field efficiency of 55%.

In order to compute the number of heliostats, for an average power of around 20 MW we take Power in Eq (S1) equal to 25 MW [1]. These plants are expected to operate 6450 h a year. Considering the annual radiation during the sun hours, we calculate the area needed in the heliostat field, given by Eq (S2).

$$Power \cdot \mathrm{Operating} = \mathrm{area}_{\mathrm{sup}} \cdot \mathrm{rad}_{annual}; \quad \text{(S1)}$$

$$\mathrm{area}_{\mathrm{sup}} = N_{\mathrm{helios}} \cdot \mathrm{Area}_{\mathrm{helio}} \cdot \eta_{\mathrm{helio}} ; \quad \text{(S2)}$$

With that field of heliostats, for each month, we have the capability of gathering a certain amount of energy given by Eq (S3). Note that, for the sake of simplicity in the presentation of the equations, from this point on we are not presenting the "time period" index in each one of them.

$$\text{radiation}\cdot N_{\text{days}}\cdot \text{area}_{\text{sup}}\cdot \text{rend}_{\text{field}} = \text{Energy}; \tag{S3}$$

The energy collected in the heliostat field is only produced during the sun hours of a day. Thus, the energy available at the collector is given by Eq (S4):

$$Q_{(\text{Colector})} = \frac{\text{Energy}}{\left(\text{sun}_{hours}\cdot \text{days}\cdot 3600\right)}; \tag{S4}$$

Based on the operating conditions of the Gemasolar plant [1] the salts are fed to the collector at 290ºC and are heated up to 565 ºC using the energy collected from solar radiation. Thus the flow of molten salts is calculated as Eq (S5) during the sun hours with the heat capacity for the molten salts from Zavoico [5]. The flow of the salts in and out of the collector is the same, Eq (S6):

$$Q_{(\text{Colector})} = fc_{(\text{salts,Colector,Tank1})}\cdot \int_{T_{(\text{Tank2,Colector})}}^{T_{(\text{Colector,Tank1})}} cp_{salt}\,dT \tag{S5}$$

$$fc_{(\text{salts,Tank2,Colector})} = fc_{(\text{salts,Colector,Tank1})}; \tag{S6}$$

The salts flow rate in the Power cycle should be lower or equal to the maximum flow generated at the collector with a correction factor given by the ratio between the sun hours in a day and the continuous operation of the plant, as presented in Eq (S7):

$$fc_{(\text{salts,Tank1,Spl1})} \leq \frac{\left(\text{sun}_{hours}\right)}{24}\cdot fc_{(\text{salts,Tank2,Colector})}; \tag{S7}$$

We assume that there are no losses in the salts closed cycle, which is modeled using Eq (S8):

$$fc_{(\text{salts,HX3,Tank2})} = fc_{(\text{salts,Tank1,Spl1})}; \tag{S8}$$

<u>Turbine system.</u>

A regenerative Rankine cycle is typically used [2]. Figure S1 presents a scheme representing the Rankine cycle implemented.

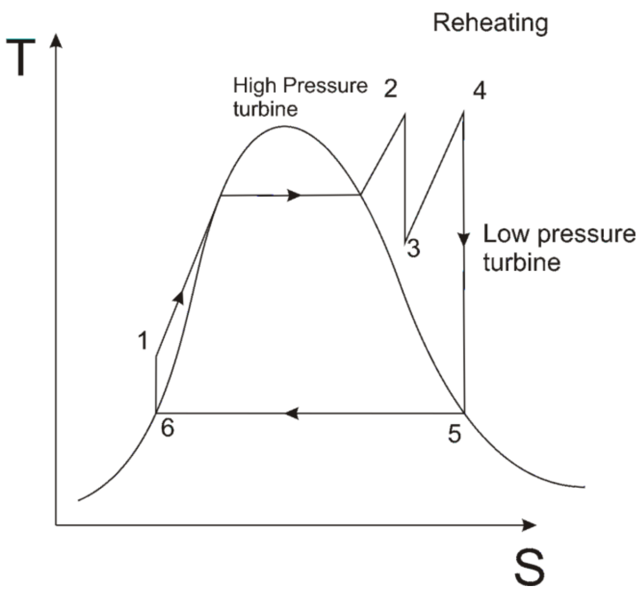

Figure S1. Rankine regenerative cycle

High pressure steam is generated using the molten salts. In the literature the inlet pressure to the first body of the turbine ranges typically from 40 to 126 bar [6-14]. Thus, we consider a range from 90 bar to 125 bar for the steam being fed to the turbine. The high pressure superheated steam is expanded into a mean pressure. We allow a range from 11 to 35 bar based on data from the literature [6, 8, 11, 12].

First we present the mass and energy balances to the set of heat exchangers that transfer the solar energy stored in the molten salts into the water/steam that follows the power cycle. In Figure S1 we follow the salts path. The flow of salts from storage tank 1 is split so that part of hot salts are used to heat up steam previous to the turbine, HX1, and the rest is used for a reheating stage, heat exchanger 4 (HX4), Eq (S9):

$$\mathrm{fc}_{(\mathrm{salts,Tank1,Spl1})} = \mathrm{fc}_{(\mathrm{salts,Spl1,HX1})} + \mathrm{fc}_{(\mathrm{salts,Spl1,HX4})}; \qquad \text{(S9)}$$

HX1 is used to heat up the saturated steam at the operating pressure of the turbine employing the hot molten salts. The entalphy and entrophy of the different water streams in the different aggregation states (compressed liquid, saturated liquid, saturated vapor and superheated steam) were correlated as function of the pressure and temperature. Thus, the energy provided by the hot molten salts, calculated as Eq (S10), corresponds to the energy transferred to the steam in HX1 as Eq (S11):

$$\mathrm{Q}_{(\mathrm{HX1})} = -\mathrm{fc}_{(\mathrm{salts,Spl1,HX1})} \int_{\mathrm{T}_{(\mathrm{Spl1,HX1})}}^{\mathrm{T}_{(\mathrm{HX1,Mix1})}} cp_{salt} dT \qquad \text{(S10)}$$

$$Q_{(HX1)} = fc_{(Wa,HX2,HX1)} \cdot \left(H_{steam,(HX1,Turbine1)} - H_{steam,(HX2,HX1)} \right); \tag{S11}$$

To ensure saturation conditions, the temperature at the inlet of heat echanger 1, HX1, is that given by Antoine equation (S12):

$$P_{turb1} \cdot 760 = e^{\left(A(Wa) - \frac{B(Wa)}{\left(C(Wa) + T_{(HX2,HX1)} \right)} \right)} \tag{S12}$$

For each heat exchanger in the system we allow that aminimum approach tempearture of 10ºC. The salts leaving HX1 are mixed with hot salts from Tank 1. The mixing is modeled using Eq (S13)-(S16) .

$$fc_{(salts,HX1,Mix1)} = fc_{(salts,Spl1,HX1)}; \tag{S13}$$

$$fc_{(salts,HX1,Mix1)} + fc_{(salts,HX4,Mix1)} = fc_{(salts,Mix1,HX2)}; \tag{S14}$$

$$fc_{(salts,Spl1,HX4)} = fc_{(salts,HX4,Mix1)}; \tag{S15}$$

$$fc_{(salts,HX1,Mix1)} \int_{T_{(HX1,Mix1)}}^{T_{(Mix1,HX2)}} cp_{salt} dT + fc_{(salts,HX4,Mix1)} \int_{T_{(HX4,Mix1)}}^{T_{(Mix1,HX2)}} cp_{salt} dT = 0 \tag{S16}$$

The flow of salts from Mix1 is used to evaporate the saturated water coming from HX3 in HX2. HX2 is modelled using eqs. (S17)-(S20):

$$Q_{(HX2)} = -fc_{(salts,Mix1,HX2)} \cdot \int_{T_{(Mix1,HX2)}}^{T_{(HX2,HX3)}} cp_{salt} dT = 0 \tag{S17}$$

$$Q_{(HX2)} = fc_{(Wa,HX3,HX2)} \cdot \left(H_{steam,(HX2,HX1)} - H_{liq,(HX3,HX2)} \right); \tag{S18}$$

$$fc_{(Wa,HX3,HX2)} = fc_{(Wa,HX2,HX1)}; \tag{S19}$$

$$fc_{(salts,HX2,HX3)} = fc_{(salts,HX3,Tank2)} \tag{S20}$$

The salts leaving HX2 are used in HX3 to heat up the compressed liquid stream already at P_{turb1} from HX6. HX3 is modelled using mass and energy balance gien by eqs (S21)-(S24):

$$Q_{(HX3)} = -fc_{(salts,HX2,HX3)} \int_{T_{(HX2,HX3)}}^{T_{(HX3,Tank2)}} cp_{salt} dT \tag{S21}$$

$$Q_{(HX3)} = fc(Wa, HX6, HX3)\cdot\left(H_{liq,(HX3,HX2)} - H_{liq,(HX6,HX3)} \right) \quad (S22)$$

$$fc_{(Wa,HX6,HX3)} = fc_{(Wa,HX3,HX2)}; \quad (S23)$$

$$fc_{(Wa,HX1,Turbine1)} = fc_{(Wa,HX2,HX1)}; \quad (S24)$$

The superheated steam is sent from HX1 to the first body of the turbine. We consider that the turbine consits of three bodies and all of them are modelled similarly. The expansion of the steam in the different bodies of the turbine is assumed to have an isentropic efficiency of 0.9 [15]. Therefore, the stream exiting the first body can be calculated using eqs. (S25)-(S27)

$$\eta = \frac{H_{steam,(Turbine1,HX4)} - H_{steam,(HX1,Turbine1)}}{H_{steam,(isoentropy)} - H_{steam,(HX1,Turbine1)}} \quad (S25)$$

Where

$$H_{steam,(isoentropy)} = f\left(p_{(Turbine1,HX4)}, T^{*}_{(Turbine1,HX4)} \right) \quad (S26)$$

T* represents the isoentropic temperature after the expansion. And

$$s_{steam,(HX1,Turbine1)} = f\left(p_{(HX1,Turbine1)}, T_{(HX1,Turbine1)} \right) = f(p_{(Turbine2,HX4)}, T^{*}_{(Turbine1,HX4)}) \quad (S27)$$

To enforce that the output of the turbine is superheated steam we use Eqs (S28)-(S29).

$$p_{turb2}\cdot 760 = e^{\left(A(Wa) - \frac{B(Wa)}{(C(Wa) + T_{turb1min})} \right)} \quad (S28)$$

$$T_{(Turbine1,HX4)} > T_{turb1min}; \quad (S29)$$

The energy that is obtained in the first body of the turbine is given by Eq (S30):

$$W_{(Turbine1)} = fc_{(Wa,HX1,Turbine1)}\cdot\left(H_{steam,(HX1,Turbine1)} - H_{steam(Turbine1,HX4)} \right) \quad (S30)$$

The stream as superheated vapor is heated up again in HX4 using a fraction of the total flow of molten salts stored in Tank1. Next, the superheated steam is fed to the second body of turbine. HX4 is modeled using Eq (S31)-(S32)

$$Q_{(HX4)} = fc_{(Wa,Turbine1,HX4)}\cdot\left(H_{steam,(HX4,Turbine2)} - H_{steam,(Turbine1,HX4)} \right); \quad (S31)$$

$$Q_{(HX4)} = -fc_{(salts,Spl1,HX4)} \cdot \int_{T_{(Spl1,HX4)}}^{T_{(HX4,Mix1)}} cp_{salt} dT \quad (S32)$$

In the second body of the turbine there is another expansion to a lower pressure. Part of the stream is sent to HX6 while the rest is used in the third body of the turbine where it is expanded to the exhaust pressure exiting as saturated vapor. This last pressure ranges from 0.05 bar to 0.31 bar [6,7,11,12,14] but it is typically a fixed parameter. We consider it as an optimization variable within the range of 0.05 to 0.35 bar. The stream splitter after the second body of the turbine is modelled by Eq (S33):

$$fc_{(Wa,HX4,Turbine2)} = fc_{(Wa,Turbine2,HX6)} + fc_{(Wa,Turbine2,Turbine3')}; \quad (S33)$$

and sent to HX6 where it is used to reheat the liquid obtained after condensing the exhaust of the third body of the turbine, Eq. (S34)

$$Q_{(HX5)} = fc_{(Wa,Turbine3,HX5)} \cdot \left(H_{liq,(HX5,HX6)} - H_{steam(turbine3,HX5)} \right); \quad (S34)$$

The energy from HX5 is removed from the system using a cooling tower, see section 2.1.3. The stream exiting HX6 must be liquid so that it is compressed using a pump up to P_{turb1}. To enforce this we impose Eq (S35):

$$T_{(HX6,HX3)} \leq T_{turb2min}; \quad (S35)$$

Where $T_{turb2min}$ is the saturated temperature at the pressure of the exhaust if the second body of the turbine calculated using Antonine correlation. The total energy obtained in the systems to be optimized is the sum of the ones generated at the three bodies of the turbine, Eq. (S36):

$$W_{total} = W_{(Turbine1)} + W_{(Turbine2)} + W_{(Turbine3)}; \quad (S36)$$

S2. Cost estimation equations of equipment

The equipment should be designed to operate throughout the entire year, so the maximum requirement for each design variable is considered for the cost estimation. Eq. (S37) shows the selection of the maximum value for Q_{SR}; the constraints for the rest of the equipment can be consulted in the Supporting Information, eqs. (S39)-(S45).

$$Q_{SR,l}^{des} \geq Q_{SR,t,l} \qquad \forall t \in TD, \forall l \in Loc \qquad \text{(S37)}$$

The correlations to estimate the cost for each equipment (i.e., heliostat field, heat exchangers, storage tanks, turbines system, solar receiver, wet-cooling, and dry-cooling) are also presented in the Supporting Information, eqs. (S46)-(S94). The investment cost of the facility ($Invest_{fac}$, €$_{2025}$) can be obtained as the sum of each independent cost and multiply by 6 [16], as seen in eq. (S38):

$$Invest_{fac,l} = 6 \cdot \sum_{i=1}^{eq} Cost_{i,l}^{des} \qquad \forall l \in Loc \qquad \text{(S38)}$$

Where $Cost_{i}^{des}$ is the cost of the i-equipment/element, that must be contained in the set of units or elements of the facility $eq = \{WC, DC, SR, tank1, tank2, Turb, HX1, HX2, HX3, HX4, ground, hel\}$. Each cost is scaled from their original currency ($ or €) to €$_{2025}$, considering the effect of the inflation rate.

Constraints of cost estimation

The constraints presented in eqs. (S39)-(S45) are defined to consider the design point of each equipment:

$$m_{tank2-SR,l}^{des} \geq m_{tank2-SR,t,l} \qquad \forall t \in TD, \forall l \in Loc \qquad \text{(S39)}$$

$$Q_{cool,l}^{des} \geq Q_{cool,t,l} \qquad \forall t \in TD, \forall l \in Loc \qquad \text{(S40)}$$

$$Q_{HX1,l}^{des} \geq Q_{HX1,t,l} \qquad \forall t \in TD, \forall l \in Loc \qquad \text{(S41)}$$

$$Q_{HX2,l}^{des} \geq Q_{HX2,t,l} \qquad \forall t \in TD, \forall l \in Loc \qquad \text{(S42)}$$

$$Q_{HX3,l}^{des} \geq Q_{HX3,t,l} \qquad \forall t \in TD, \forall l \in Loc \qquad \text{(S43)}$$

$$Q_{HX4,l}^{des} \geq Q_{HX4,t,l} \qquad \forall t \in TD, \forall l \in Loc \qquad \text{(S44)}$$

$$W_{Turb,l}^{des} \geq W_{Turb,t,l} \qquad \forall t \in TD, \forall l \in Loc \qquad \text{(S45)}$$

Solar receiver and heliostat field

The cost of solar receiver ($Cost_{SR}$, €2020) and heliostat field ($Cost_{field}$, €2020) can be calculated using eqs. (S46)-(S47) [17]:

$$Cost_{SR,l} = 200 \cdot W^{des}_{Turb,l} \qquad \forall l \in Loc \qquad \text{(S46)}$$

$$Cost_{field,l} = 120 \cdot A_{hel} + A_{layout,l} \cdot Cost_{ground,l} \qquad \forall l \in Loc \qquad \text{(S47)}$$

The values of cost of ground for each province ($Cost_{ground}$, €2019/m^2), for the case of study, are collected from Spanish Ministry of Agriculture surveys [18].

Heat exchangers

In order to simplify the nomenclature of this subsection, heat exchangers (HXs) equations are written using the set of heat exchangers $e \in [1,2,3,4]$, where the e-number corresponds with its position in the region. The design cost of heat exchangers ($Cost^{des}_{HXe}$, €2020) requires knowing previously the value of the heat exchanger area ($Area^{des}_{HXeq}$, m^2), which can be determined by eq. (S48):

$$Q^{des}_{HXe,l} = Area^{des}_{HXe,l} \cdot U_{HXe,l} \cdot LMTD_{HXe,l} \qquad \forall l \in Loc \qquad \text{(S48)}$$

Where U_{HXeq} is the global heat transfer coefficient of the equipment (kW/(m^2 K)), and $LMTD_{HXeq}$ is the logarithm mean temperature difference of the equipment (K). The values of $LMTD_{HXeq}$ are the same for every location and can be fixed according to previous works [19]; those data are collected in Table S1. As the design of the equipment is out of the scope of this work, a mean U_{HXel} is estimated following previous works (0.16 kW/(m^2·K)) [20].

Table S1: Values of LMTD.

**Equipment**	**HX1**	**HX2**	**HX3**	**HX4**

**LMTD (K)**	40	55	50	47

The area $Area_{HXe}^{des}$ is employed to estimate the cost of the heat exchanger ($Cost_{HXe,l}$, €2020), as seen in eq. (S49) for areas larger than 140 m² [17]:

$$Cost_{HXe,l} = 22.234 \cdot \left(A_{HXe,l}^{des}\right)^{04671} \qquad \forall l \in Loc \qquad \text{(S49)}$$

To avoid non-linearities in the model, eq. (S11) is approximated as a linear regression in the interval of interest, as seen in eq. (S48). Furthermore, to consider the existence of the facility and calculate the design cost ($Cost_{HXe}^{des}$, €2020), a Big-M formulation is applied employing y_{ff}, as presented in eqs. (S50)-(S53):

$$Cost_{HXe,l} = 478.01 \cdot Area_{HXe,l}^{des} + 167{,}377 \qquad \forall l \in Loc \qquad \text{(S50)}$$

$$Cost_{HXe,l}^{des} \geq Cost_{HXe,l} - \left(1 - y_{ff,l}\right) \cdot BigM \qquad \forall l \in Loc \qquad \text{(S51)}$$

$$Cost_{HXe,l}^{des} \leq y_{ff,l} \cdot BigM \qquad \forall l \in Loc \qquad \text{(S52)}$$

$$Cost_{HXe,l}^{des} \leq Cost_{HXe,l} \qquad \forall l \in Loc \qquad \text{(S53)}$$

<u>Dry-cooling system</u>

The estimation of the cost of dry-cooling system ($Cost_{DC}$, \$2014) is carried out employing the eq. (S54) [21]:

$$Cost_{DC,l} = 7{,}591.5 \cdot \left(A_{DC,l}^{des}\right)^{0.396} \qquad \forall l \in Loc \qquad \text{(S54)}$$

Eq. (S16) is also redefined using piecewise-Linear approximation [22] and BigM formulation in order to eliminate non-linearities, as seen in eqs. (S55)-(S58):

$$Cost_{DC,l} = 14.922 \cdot Area_{DC,l}^{des} + 135{,}540 \qquad \forall l \in Loc \qquad \text{(S55)}$$

$$Cost_{DC,l}^{des} \geq Cost_{DC,l} - \left(1 - y_{DC,l}\right) \cdot BigM \qquad \forall l \in Loc \qquad \text{(S56)}$$

$$Cost_{DC,l}^{des} \leq y_{DC,l} \cdot BigM \qquad \forall l \in Loc \qquad \text{(S57)}$$

$$Cost_{DC,l}^{des} \leq Cost_{DC,l} \qquad \forall l \in Loc \qquad \text{(S58)}$$

Wet-cooling systems

The cost of wet-cooling systems, for each time period and province ($Cost_{WC,l}$, $2014), is estimated using the eq. (S59) [23]. The cost of the designed equipment is selected using the expression of eqs. (S60)-(S62):

$$Cost_{WC,l} = 24.043 \cdot Q_{cool,l}^{des} + 553{,}487 \qquad \forall l \in Loc \qquad \text{(S59)}$$

$$Cost_{WC,l}^{des} \geq Cost_{WC,l} - \left(1 - y_{WC,l}\right) \cdot BigM \qquad \forall l \in Loc \qquad \text{(S60)}$$

$$Cost_{WC,l}^{des} \leq y_{WC,l} \cdot BigM \qquad \forall l \in Loc \qquad \text{(S61)}$$

$$Cost_{WC,l}^{des} \leq Cost_{WC,l} \qquad \forall l \in Loc \qquad \text{(S62)}$$

Storage tanks

For the cost estimation of storage tanks, it is required to know beforehand the storage volume ($V_{storage}$, m[3]). Considering V_{MS} as the volume of molten salts that must be stored at the same time (m[3]), there are introduced the eqs. (S63)-(S64) to calculate the volumes per province and time, and the design storage volume ($V_{storage}^{des}$,m[3]):

$$V_{MS,t,l} = \frac{m_{tank2-SR,t,l} \cdot H_{sun,t,.l} \cdot 3600}{\rho_{MS}} \qquad \forall t \in TD, \forall l \in Loc \qquad \text{(S63)}$$

$$V_{storage,l}^{des} \geq V_{MS,t,l} \qquad \forall l \in Loc \qquad \text{(S64)}$$

After determining $V_{storage}^{des}$, the cost of a single tank ($Cost_{SinST,l}$, €2020) is estimated using a reformulated model for the literature correlation, eq. (S65) [17], which is shown in eqs. (S66)-(S70). The reformulated model is based on the piecewise-Linear approximation, eq. (S66), and BigM formulation in order to eliminate non-linearities, as seen on eqs. (S67)-(S69). The eq. (SI.70) shows the relation between the cost of the cold molten salt storage tank ($Cost_{tank2}^{des}$, €2020) and the hot one ($Cost_{tank1}^{des}$, €2020):

$$Cost_{SinST,l} = 6{,}839.8 \cdot \left(V_{storage,l}^{des}\right)^{0.65} \qquad \forall l \in Loc \qquad \text{(S65)}$$

$$Cost_{SinST,l} = 276.38 \cdot V_{storage,l}^{des} + 363{,}463 \qquad \forall l \in Loc \qquad \text{(S66)}$$

$$Cost_{tank1,l}^{des} \geq Cost_{SinST,l} - \left(1 - y_{ff,l}\right) \cdot BigM \qquad \forall l \in Loc \qquad \text{(S67)}$$

$$Cost^{des}_{tank1,l} \leq y_{ff,l} \cdot BigM \qquad \forall l \in Loc, \forall n \in Tk \qquad \text{(SI.68)}$$

$$Cost^{des}_{tank1,l} \leq Cost_{SinST,l} \qquad \forall l \in Loc, \forall n \in Tk \qquad \text{(SI.69)}$$

$$Cost^{des}_{tank1,l} = Cost^{des}_{tank2,l} \qquad \forall l \in Loc \qquad \text{(SI.70)}$$

Turbines system

The cost of the turbines system ($Cost_{Turb}$, €2020) is estimated using the eq. (S71) [17]:

$$Cost_{Turb,l} = 633{,}000 \cdot \left(W^{des}_{Turb,l}/1000\right)^{0.398} \qquad \forall l \in Loc \qquad \text{(S71)}$$

As previous equipment, the eq. (S71) was redefined to eliminate non-linearities, as seen in eqs. (S72)-(S94), in order to calculate the design turbines system cost ($Cost^{des}_{Turb,l}$, €2020):

$$Cost^{a}_{Turb,l} = 100{,}470 \cdot \left(W^{des}_{Turb,l}/1000\right) + 624{,}248 \qquad \forall l \in Loc \qquad \text{(S72)}$$

$$Cost^{b}_{Turb,l} = 34{,}149 \cdot \left(W^{des}_{Turb,l}/1000\right) + 1{,}000{,}000 \qquad \forall l \in Loc \qquad \text{(S73)}$$

$$Cost^{c}_{Turb,l} = 13{,}296 \cdot \left(W^{des}_{Turb,l}/1000\right) + 3{,}000{,}000 \qquad \forall l \in Loc \qquad \text{(S74)}$$

$$Cost^{d}_{Turb,l} = 7{,}146.1 \cdot \left(W^{des}_{Turb,l}/1000\right) + 4{,}000{,}000 \qquad \forall l \in Loc \qquad \text{(S75)}$$

$$b^{a}_{Turb,l} + b^{b}_{Turb,l} + b^{c}_{Turb,l} + b^{d}_{Turb,l} = 1 \qquad \forall l \in Loc \qquad \text{(S76)}$$

$$\left(W^{des}_{Turb,l}/1000\right) \geq b^{b}_{Turb,l} \cdot 10 + b^{c}_{Turb,l} \cdot 50 + b^{d}_{Turb,l} \cdot 250 \qquad \forall l \in Loc \qquad \text{(S77)}$$

$$\left(W^{des}_{Turb,l}/1000\right) \leq b^{a}_{Turb,l} \cdot 10 + b^{b}_{Turb,l} \cdot 50 + b^{c}_{Turb,l} \cdot 250 + b^{d}_{Turb,l} \cdot 15{,}000 \qquad \forall l \in Loc \qquad \text{(S78)}$$

$$\varphi^{a}_{Turb,l} \geq Cost^{a}_{Turb,l} - \left(1 - b^{a}_{Turb,l}\right) \cdot BigM \qquad \forall l \in Loc \qquad \text{(S79)}$$

$$\varphi^{a}_{Turb,l} \leq Cost^{a}_{Turb,l} \qquad \forall l \in Loc \qquad \text{(S80)}$$

$$\varphi^{a}_{Turb,l} \leq b^{a}_{Turb,l} \cdot BigM \qquad \forall l \in Loc \qquad \text{(S81)}$$

$$\varphi^{b}_{Turb,l} \geq Cost^{b}_{Turb,l} - \left(1 - b^{b}_{Turb,l}\right) \cdot BigM \qquad \forall l \in Loc \qquad \text{(S82)}$$

$$\varphi^{b}_{Turb,l} \leq b^{b}_{Turb,l} \cdot BigM \qquad \forall l \in Loc \qquad \text{(S83)}$$

$$\varphi^{b}_{Turb,l} \leq Cost^{b}_{Turb,l} \qquad \forall l \in Loc \qquad \text{(S84)}$$

$$\varphi^{c}_{Turb,l} \geq Cost^{c}_{Turb,l} - \left(1 - b^{c}_{Turb,l}\right) \cdot BigM \qquad \forall l \in Loc \qquad \text{(S85)}$$

$$\varphi^{c}_{Turb,l} \leq b^{c}_{Turb,l} \cdot BigM \qquad \forall l \in Loc \qquad \text{(S86)}$$

$$\varphi^{c}_{Turb,l} \leq Cost^{c}_{Turb,l} \qquad \forall l \in Loc \qquad \text{(S87)}$$

$$\varphi^{d}_{Turb,l} \geq Cost^{d}_{Turb,l} - \left(1 - b^{d}_{Turb,l}\right) \cdot BigM \qquad \forall l \in Loc \qquad \text{(S88)}$$

$$\varphi^{d}_{Turb,l} \leq b^{d}_{Turb,l} \cdot BigM \qquad \forall l \in Loc \qquad \text{(S89)}$$

$$\varphi^{d}_{Turb,l} \leq Cost^{d}_{Turb,l} \qquad \forall l \in Loc \qquad \text{(S90)}$$

$$Cost_{Turb,l} = \varphi^{a}_{Turb,l} + \varphi^{b}_{Turb,l} + \varphi^{c}_{Turb,l} + \varphi^{d}_{Turb,l} \qquad \forall l \in Loc \qquad \text{(S91)}$$

$$Cost^{des}_{Turb,l} \geq Cost_{Turb,l} - \left(1 - y_{ff,l}\right) \cdot BigM \qquad \forall l \in Loc \qquad \text{(S92)}$$

$$Cost^{des}_{Turb,l} \leq y_{ff,l} \cdot BigM \qquad \forall l \in Loc \qquad \text{(S93)}$$

$$Cost^{des}_{Turb,l} \leq Cost_{Turb,l} \qquad \forall l \in Loc \qquad \text{(S94)}$$

S3. Social impact of the facility location

Previous works formulated this social impact (SI, €$_{2025}$) according to 3 contributions [24]. In this work, the expression employed is shown in eq. (S95):

$$SI = F_{DR} \cdot (Social_{DR}) + F_{UR} \cdot (Social_{UR}) + F_{RP} \cdot (Social_{RP}) \qquad \text{(S95)}$$

Where $Social_{DR}$, $Social_{UR}$ and $Social_{RP}$ are the contributions of social development ratio (DR), the unemployment ratio (UR) and the relative population (RP), respectively (€$_{2025}$), meanwhile F_{DR}, F_{UR} and F_{RP} are its corresponding decision weights; these weights are normally considered to be proportional and $F_{DR} = 1$, $F_{UR} = F_{RP} = 0.5$ [25], because both terms UR and RP consider the effect of employment. Based on previous work [24], the social impact equations were adapted, as presented in eqs. (S96) - (S98):

$$(Social_{DR}) = \sum_{l=1}^{Loc} \left[\left(\frac{\{Max\ GDP\} - GDP_l}{\{Max\ GDP\}} \right) \cdot Invest_{fac,l} \right] \cdot \frac{1}{LE_{fac}} \qquad \text{(S96)}$$

$$(Social_{UR}) = \sum_{l=1}^{Loc} \left[\left(\frac{UV_l - \{Min\ UV\}}{\{Min\ UV\}} \right) \cdot W^{des}_{Turb,l} \cdot J_{Solar} \cdot Salary_l \right] \qquad \text{(S97)}$$

$$(Social_{RP}) = \sum_{l=1}^{Loc}\left[\left(\frac{\{Max\ \rho_{inhab}\} - \rho_{inhab,l}}{\{Max\ \rho_{inhab}\}}\right)\cdot W_{Turb,l}^{des}\cdot J_{Solar}\cdot Salary_l\right] \quad \text{(S98)}$$

Where GDP is the gross development per habitant (€$_{2025}$/hab), LE_{fac} is the facility life span (35 years), UV is the unemployment value (hab), $W_{Turb,l}^{des}$ is the power produced by turbines at the design point (MW), J_{Solar} is the number of direct jobs generated by the deployment of the facility (for thermal plants the value considered is 1 Job/MW) [41], ρ_{inhab} is the population density (hab/km^2), and $Salary$ is the annual salary per employee in each region (€$_{2025}$/Job). The data related to these social variables are collected from different specialized national databases [26–28].

S4. Environmental impact of water consumption

The water consumption (Wa_{con}, L/month) can be calculated considering that the value of W_{net} is related to WC scenario ($y_{WC,l} = 1$), and the values of Wa_{req}, as seen in eq. (S99):

$$Wa_{con,t,l} = W_{net,t,l}\cdot days_t\cdot OP\cdot Wa_{req,t,l} \qquad \forall t\in TD, \forall l\in Loc \quad \text{(S99)}$$

Where $days$ is the number of days, OP is the operation time (24 h). This water consumption must be lower or equal to the water available at the location, which is calculated considering that only a fraction of the water of the region is accessible for CSP plants (FWA), as seen in eq. (S100). In this work, it is considered that only 10% of available water can be employed for CSP operation ($FWA = 0.10$).

$$Wa_{con,t,l} \le Wa_{ava,t,l}\cdot FWA \qquad \forall t\in TD, \forall l\in Loc \quad \text{(S100)}$$

To capture the issues related to water consumption in arid regions, the water index WX is introduced. This index represents the annual mean impact that the water consumption denotes, and it is calculated in 3 steps: 1) the minimum water required per person and day is fixed; 2) combining the minimum water required and the population of each region, the monthly regional water required is calculated, and also the water availability to meet 1 year of water demand; and 3) the effective water availability is estimated subtracting

the previous value to each month, and the water index WX is calculated using (S101), where $\{Max\ EWA\}$ is the maximum effective water availability in location l at period t (hm³).

$$WX_l = \frac{1}{TD} \cdot \sum_{t=1}^{TD} \frac{\{Max\ EWA\}_{t,l} - Wa_{con,t,l}}{\{Max\ EWA\}_{t,l}} \qquad \forall l \in Loc \qquad \text{(S101)}$$

Water consumption per person may involve daily water use or water related to societal processes (e.g., food production, cleaning, etc.). According to the Sphere community standards for humanitarian action, there is a consensus that the minimum amount of guaranteed water during emergencies should be 17.5 L [29], and an amount of 100 L is recommended for everyday life [30]. In this work it is considered 100 L per person and day (approximately $3{\cdot}10^{-6}$ hm^3/(hab·month)) as the minimum water required, so $\{Max\ EWA\}$ is calculated as seen in eq. (S102):

$$\{Max\ EWA\}_{t,l} = Wa_{ava,t,l} - 3 \cdot 10^{-6} \cdot Population_l \qquad \forall t \in TD, \forall l \in Loc \qquad \text{(S102)}$$

There are defined a constraint to force the selection of dry cooling if there is not enough water, eq. (S103):

$$\left(\{Max\ EWA\}_{t,l} \leq 0 \ \vee \ WX_l < 0\right) \Rightarrow \left(y_{wc,l} = 0\right) \qquad \forall t \in TD, \forall l \in Loc \qquad \text{(S103)}$$

S5. Location related data

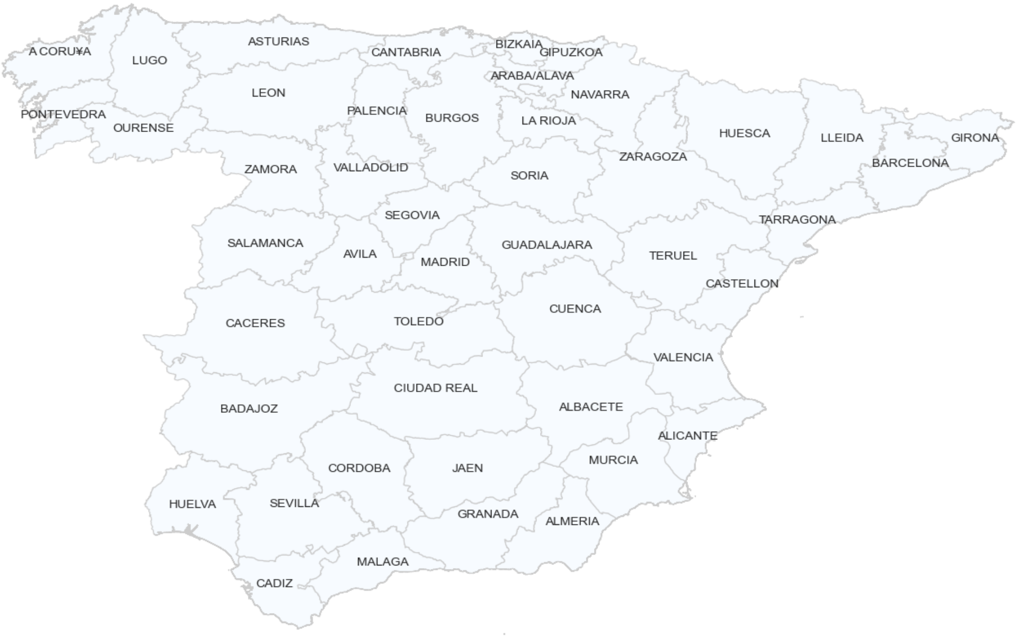

Figure S2: Distribution of Spanish provinces.

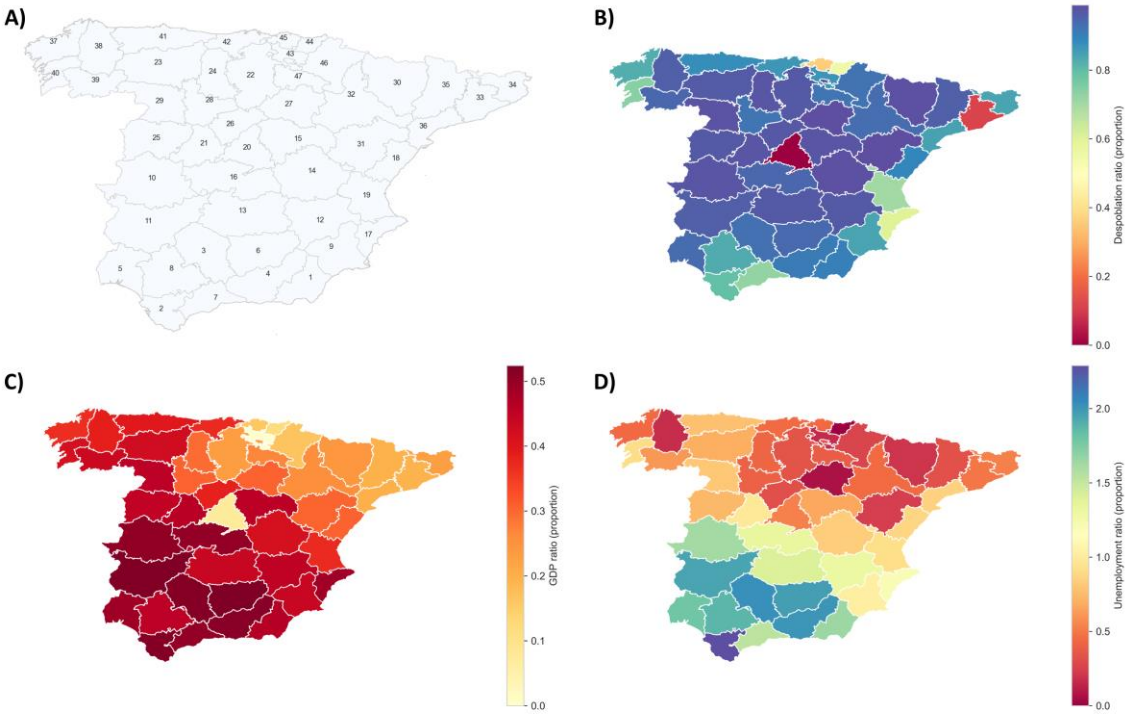

Figure S3: General information about Spain: A) discretization per province; B) population density per province; C) GDP per province; D) unemployment ratio per province.

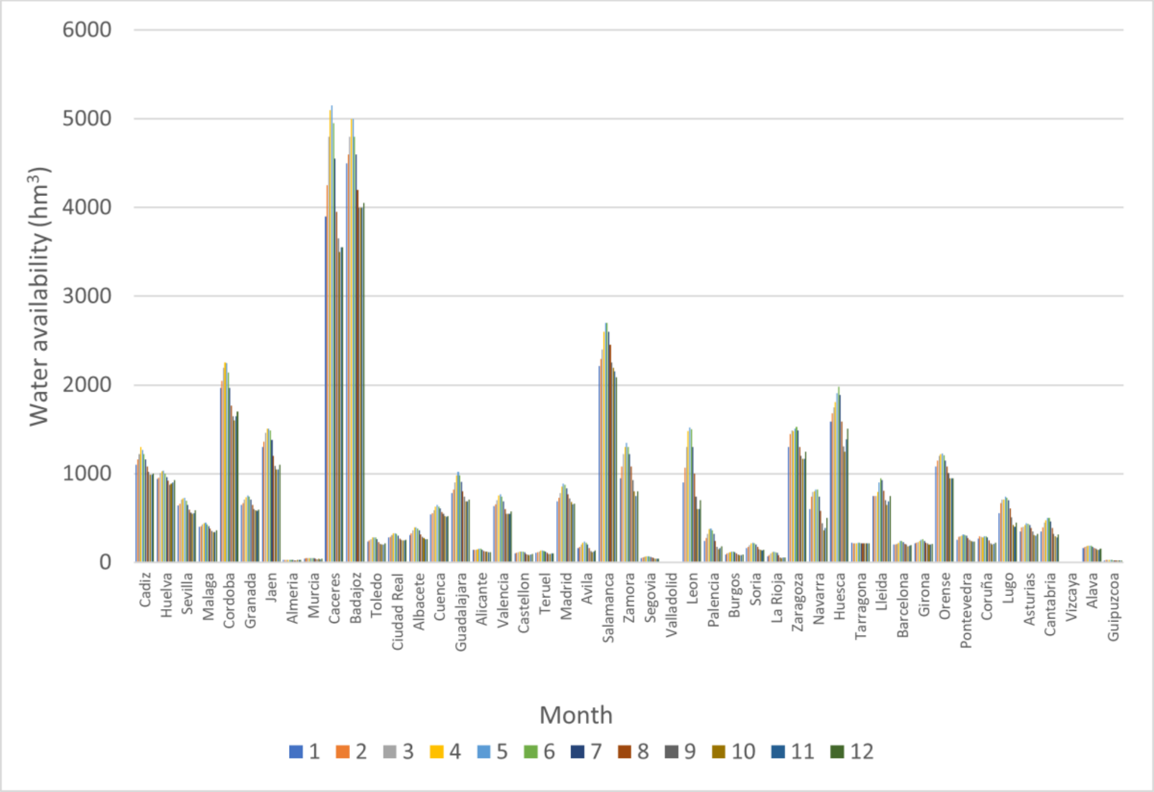

Figure S4: Total water availability per region and month in Spain [31].

Table S2: Calculation of water requirements for the population.

Region	Population (annual mean)	Water required (hm^3/month)
Alava	323,448	0.97
Albacete	395,045	1.19
Alicante	1,885,920	5.66
Almeria	703,840	2.11
Avila	165,397	0.50
Badajoz	684,430	2.05
Barcelona	5,566,863	16.70
Burgos	365,981	1.10
Caceres	405,089	1.22
Cadiz	1,238,831	3.72
Castellon	590,440	1.77
Ciudad Real	513,553	1.54
Cordoba	794,429	2.38
Coruña	1,131,727	3.40
Cuenca	206,837	0.62
Girona	759,474	2.28
Granada	916,133	2.75
Guadalajara	254,584	0.76
Guipuzcoa	715,426	2.15
Huelva	519,613	1.56
Huesca	224,279	0.67
Jaen	653,494	1.96
Leon	479,968	1.44
Lleida	436,842	1.31

Region	Population (annual mean)	Water required (hm^3/month)
La Rioja	319,259	0.96
Lugo	341,275	1.02
Madrid	6,517,091	19.55
Malaga	1,634,367	4.90
Murcia	1,473,039	4.42
Navarra	643,473	1.93
Ourense	320,945	0.96
Asturias	1,053,497	3.16
Palencia	166,767	0.50
Pontevedra	950,865	2.85
Salamanca	341,453	1.02
Cantabria	586,253	1.76
Segovia	158,780	0.48
Sevilla	1,931,811	5.80
Soria	91,791	0.28
Tarragona	804,007	2.41
Teruel	139,867	0.42
Toledo	696,150	2.09
Valencia	2,564,085	7.69
Valladolid	526,881	1.58
Vizcaya	1,152,577	3.46
Zamora	183,817	0.55
Zaragoza	964,898	2.89

Table S3: Total area per province in Spain.

Province	Total area (km^2)
Alava	3,037
Albacete	14,924
Alicante	5,817
Almeria	8,775
Avila	8,050
Badajoz	21,766
Barcelona	7,728
Burgos	14,292
Caceres	19,868
Cadiz	7,440
Castellon	6,662
Ciudad Real	19,813
Cordoba	13,771
Coruña	7,951
Cuenca	17,140
Girona	5,910
Granada	12,647
Guadalajara	12,214
Guipuzcoa	1,980
Huelva	10,128
Huesca	15,636
Jaen	13,496
Leon	15,581
Lleida	12,172

Province	Total area (km^2)
La Rioja	5,045
Lugo	9,856
Madrid	8,028
Malaga	7,306
Murcia	11,314
Navarra	10,391
Ourense	7,273
Asturias	10,604
Palencia	8,052
Pontevedra	4,495
Salamanca	12,350
Cantabria	5,321
Segovia	6,921
Sevilla	14,036
Soria	10,306
Tarragona	6,303
Teruel	14,810
Toledo	15,370
Valencia	10,776
Valladolid	8,111
Vizcaya	2,217
Zamora	10,561
Zaragoza	17,274

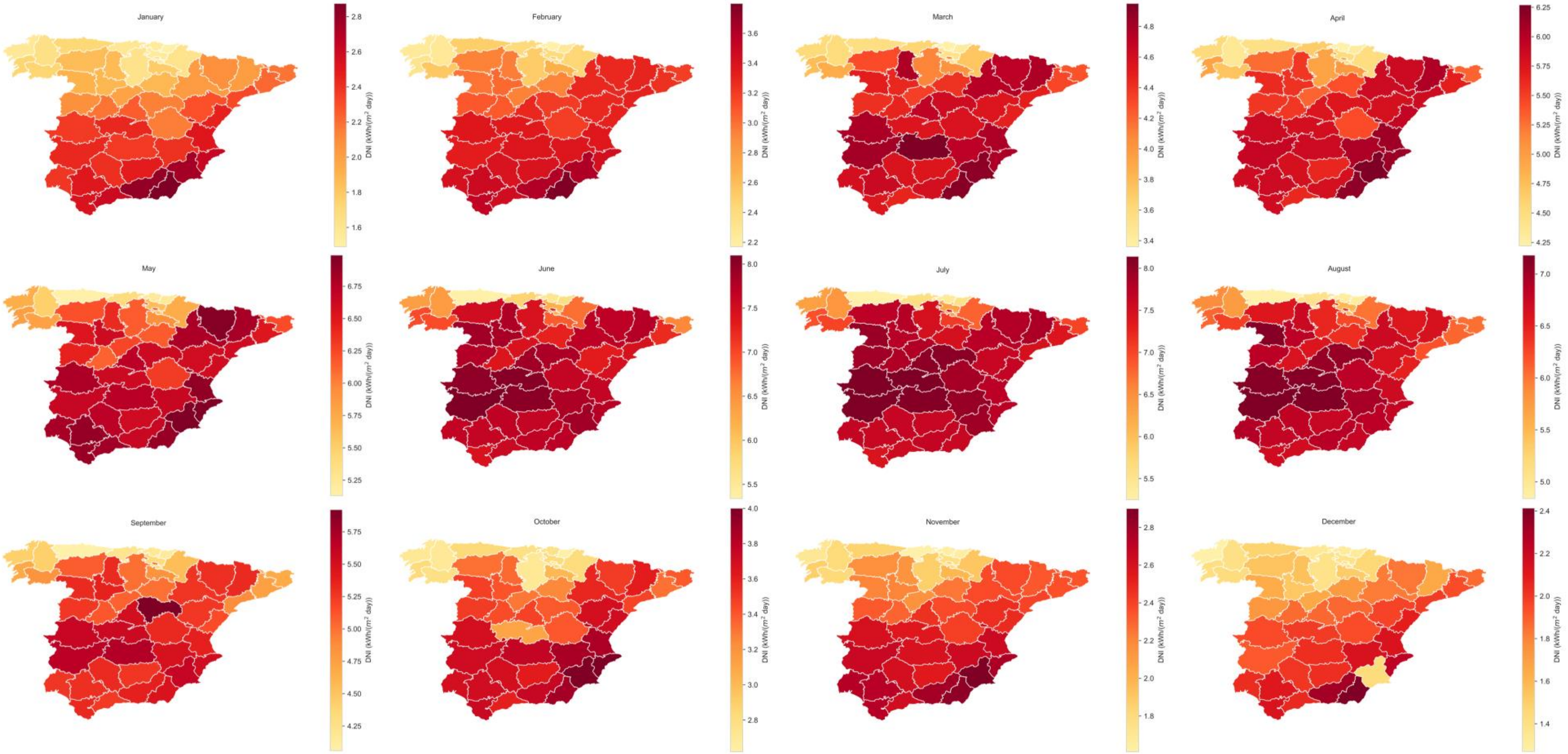

Figure S5: Direct Normal Irradiance of Spanish provinces; data from [32].

S6. Evaluation of model performance

To validate the behavior of the model on a larger scale, it is required to evaluate the results that can be obtained with a reduced set of possible locations. For the evaluation, three locations were selected throughout Spain (2, 14, and 45), which are representative in terms of different values in the characteristic parameters of each location (e.g., social indices), as shown in Table S4.

Table S4: Location-related parameters for sensitivity analysis.

Region	Province [km²]	Land Cost [€/m²]	GDP	UV	RP	WX
**2 (Cadiz)**	7,436	0.020	0.52	2.29	0.79	0.80
**14 (Cuenca)**	17,140	0.006	0.41	0.83	0.99	0.93
**45 (Bizkaia)**	2,217	0.014	0.16	0.44	0.36	1.00

Table S5: Power demand over 1 year for sensitivity analysis.

Month	Jan	Feb	Mar	Apr	May	Jun	Jul	Aug	Sep	Oct	Nov	Dec
$W_{Dem,t}$ (MW)	1.18	0.95	0.86	0.86	0.96	1.21	1.44	1.36	1.30	1.26	1.28	1.18

As the validation considers a small number of locations, the power demand to meet must be reduced on the same scale, to be able to study solutions that can be distinguished between them; Table S5 shows the demand considered. The analysis was carried out evaluating the objective function eq. (29) term by term, according to five scenarios: 1) feasibility of meeting the demand, reducing W_{add}, eq. (S104); 2) minimize the facility cost, eq. (S105); 3) evaluation of the effect of increasing the demand to be reached (10 times higher) with the additional constraint of investing at least 20 M€ in location 45; 4) maximizing SI, eq. (S106); and 5) solving the objective function, eq. (29). A total investment budget of 30M€ is considered, except for scenario 3.

$$min\left\{-\sum_{t=1}^{TD}\left[\sum_{l=1}^{Loc}\left(W_{neat,t,l}\right)+W_{add,t}\right]\right\} \quad (S104)$$

$$min\left\{\sum_{l=1}^{Loc}\left(Cost_{fac,l}\right)\cdot\frac{1}{LE_{fac}}\right\} \quad (S105)$$

$$min\,\{-SI\} \quad (S106)$$

The results for the evaluation are collected in Table S6. In general, without additional considerations, the best solution is to locate CSP plants towards the south of the country (region nº2, Cadiz). This result is

consistent with the actual trend and the fact that this is where the highest annual radiation is available [32,33]. It can be noticed that a single installation is sufficient for most of the cases, noting how the importance of the social indices alters the solution (scenarios 1 and 4 vs 5); this difference highlights the importance of policy decisions that need to be made, because it is observed that the selection of the cooling system is affected by the evaluation of the social and environmental impact, since according to the eqs. (S97)-(S98), a larger W_{Turb}^{des} implies a higher SI. Therefore, oversizing the installation to supply energy to the A-frame dry-cooling system would be socially beneficial, and the avoided water consumption reduces the environmental impact of the facility.

Table S6: Results of sensitivity analysis: scenarios 1-5.

Variable	Location	Scenarios				
		1	2	3	4	5
Number of heliostats	Cadiz	1,193	288	2,420	1,239	288
	Cuenca	0	0	0	0	0
	Bizkaia	0	0	539	0	0
Power Design (MW)	Cadiz	17.92	4.33	36.36	18.61	4.33
	Cuenca	0	0	0	0	0
	Bizkaia	0.00	0.00	6.05	0	0
Region cost (M€)	Cadiz	30.00	8.83	57.84	30.00	8.83
	Cuenca	0	0	0	0	0
	Bizkaia	0	0	20.00	0	0
Social DR (M€)	Cadiz	7.74E-01	2.28E-01	1.49E+00	7.74E-01	2.28E-01
	Cuenca	0	0	0	0	0
	Bizkaia	0	0	1.61E-01	0	0
Social UR (M€)	Cadiz	7.06E-01	1.71E-01	1.43E+00	7.33E-01	1.71E-01
	Cuenca	0	0	0	0	0
	Bizkaia	0	0	7.70E-02	0	0
Social RP (M€)	Cadiz	2.45E-01	5.92E-02	4.97E-01	2.55E-01	5.92E-02
	Cuenca	0	0	0	0	0
	Bizkaia	0	0	6.25E-02	0	0
Social Impact (M€)	Cadiz	1.25E+00	3.43E-01	2.46E+00	1.84E+00	3.43E-01
	Cuenca	0	0	0	0	0
	Bizkaia	0	0	2.30E-01	0	0
Water Impact (M€)	Cadiz	4.28E-04	0	8.68E-04	0	0
	Cuenca	0	0	0	0	0
	Bizkaia	0	0	0	0	0

S7. Additional results

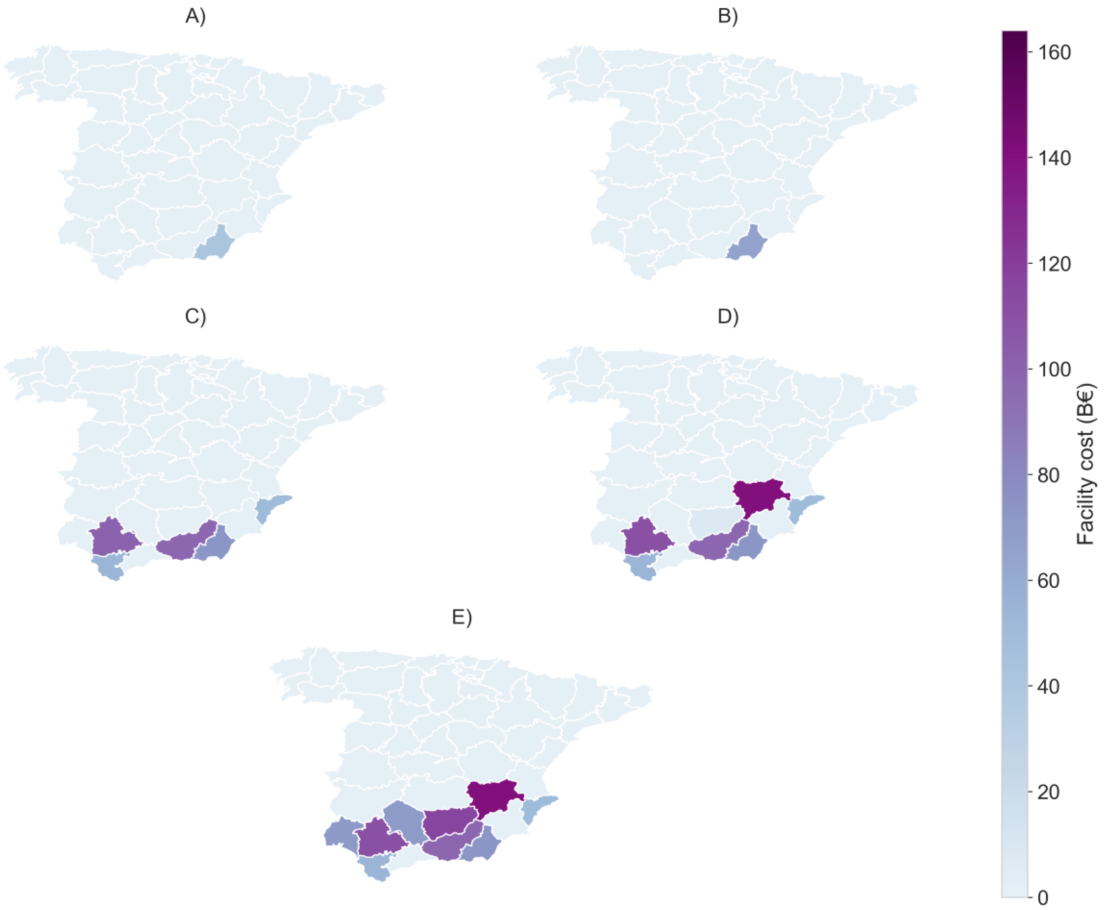

Figure S6: Investment cost for each case study scenario.

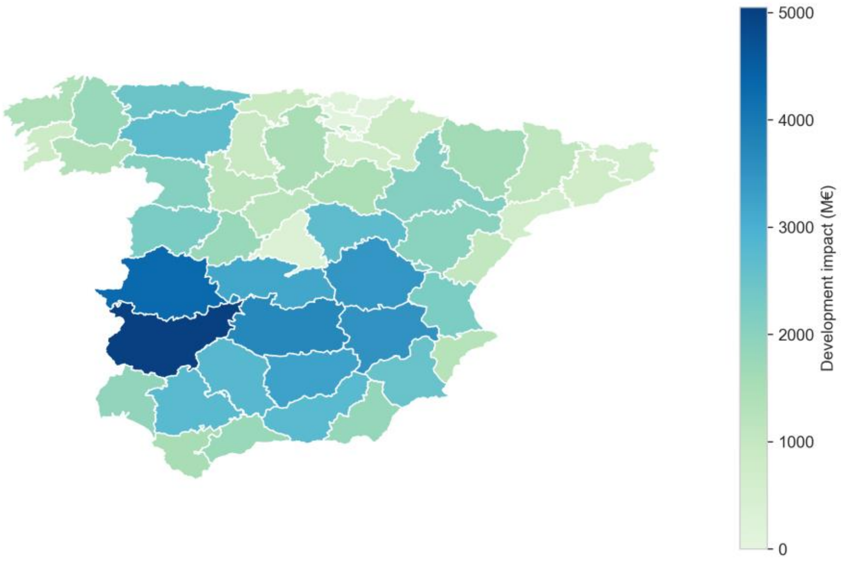

Figure S7: Maximum social impact in each Spanish province.

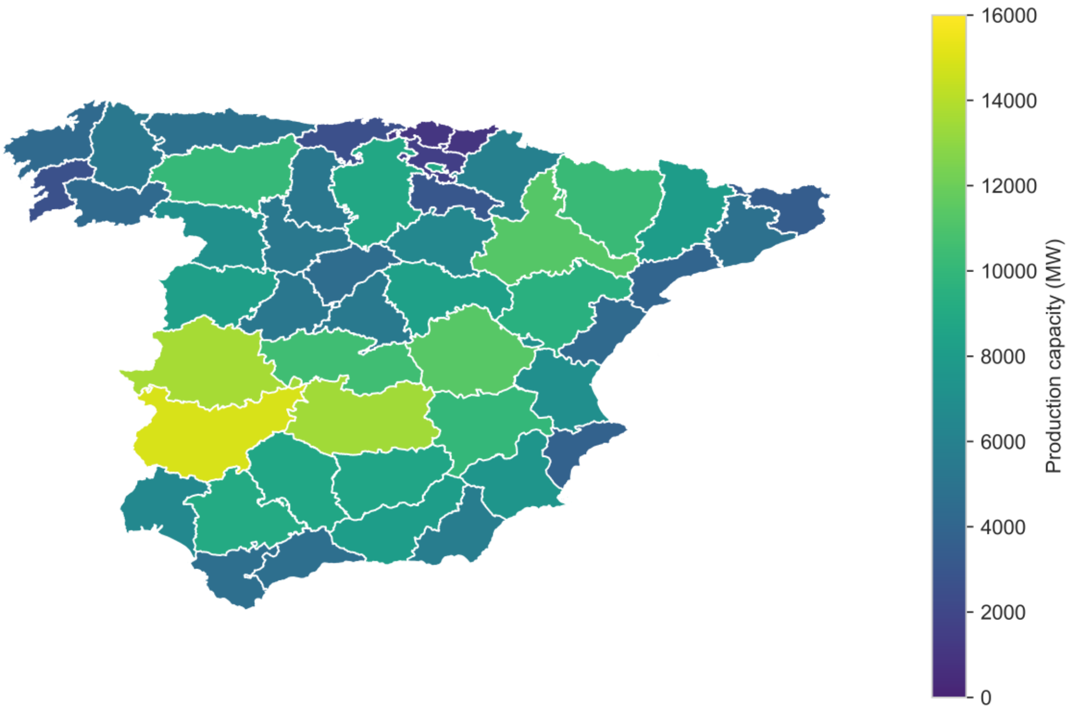

Figure S8: Maximum production capacity in each Spanish province.

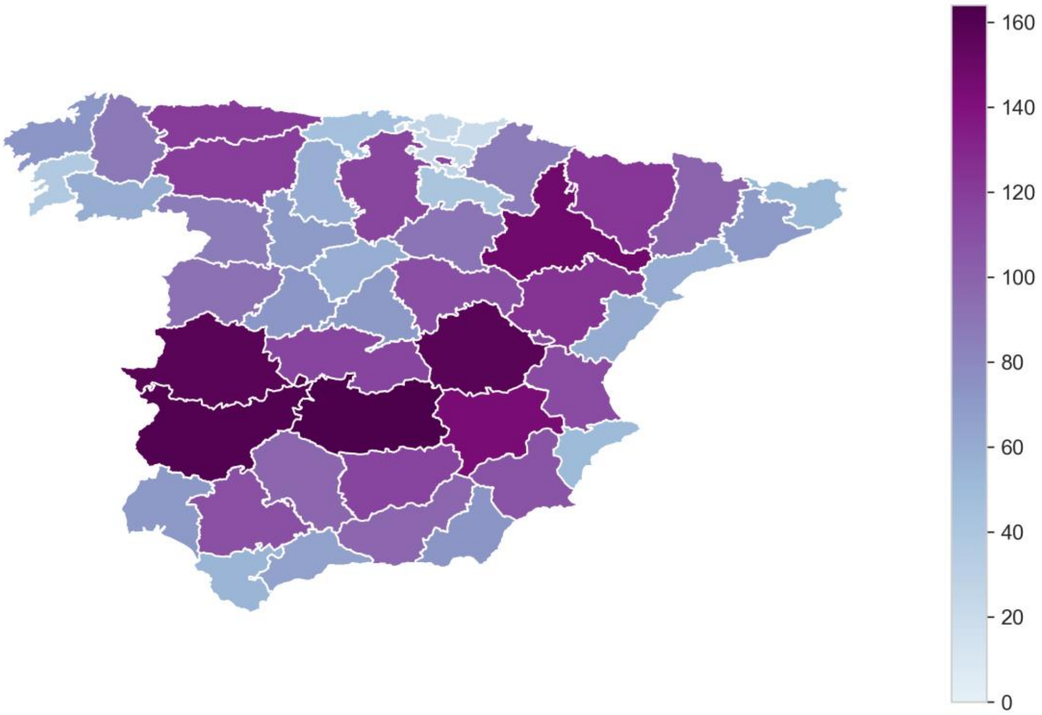

Figure S9: Maximum investment in each Spanish province.

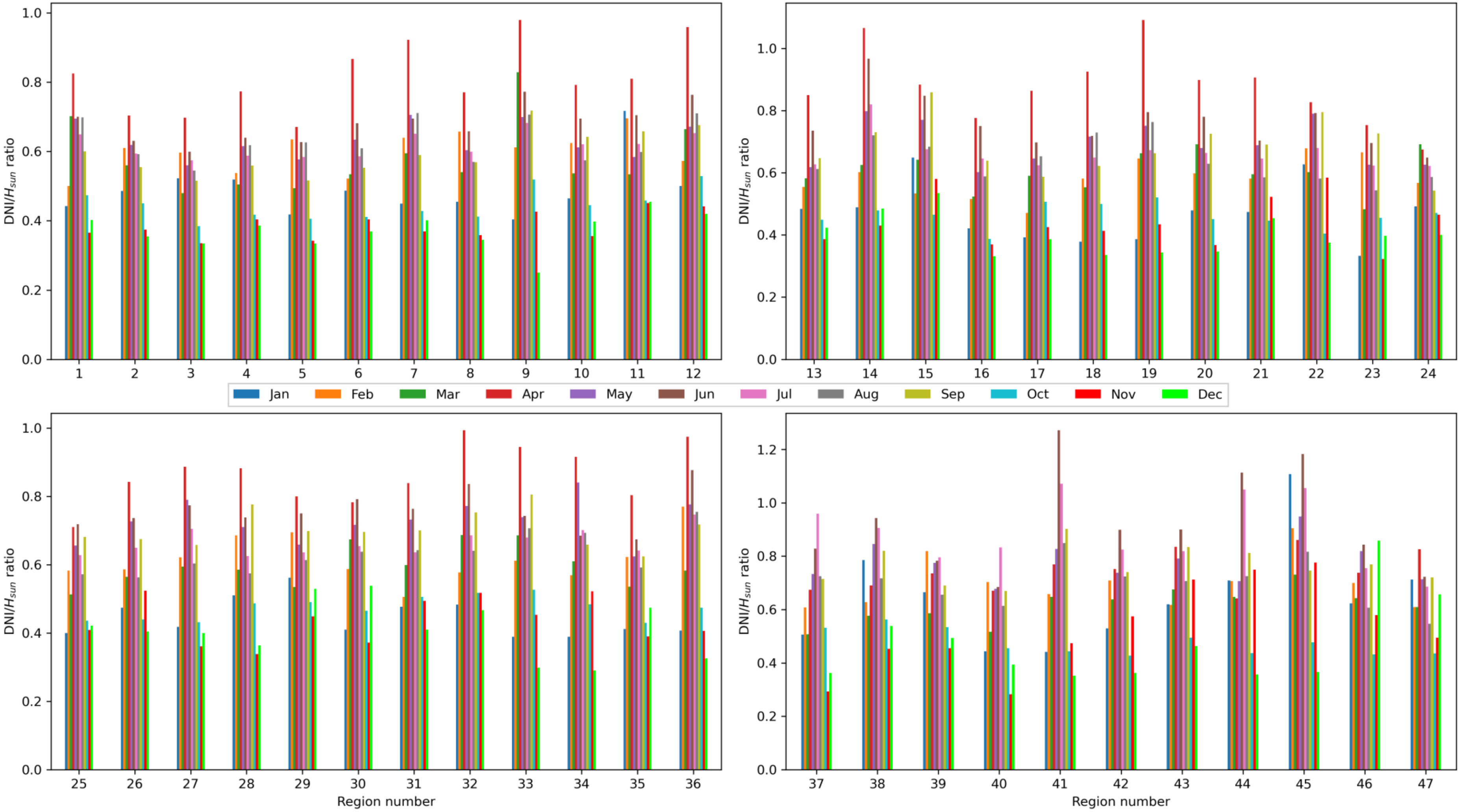

Figure S10: DNI/H_{sun} ratio for each location and month.

Table S7: Results of individual facility investment per scenario.

Energy sources considered	Facility number	Region number	Investment (M€$_{2025}$)	Fraction of total cost (%)
C	1	1	42,450	100
C + Di + TG + RNR	1	1	67,041	100
C + Di + TG + RNR + CC	1	1	72,828	19.41
	2	2	54,137	14.43
	3	4	98,725	26.31
	4	8	100,108	26.68
	5	17	50,181	13.37
C + Di + TG + RNR + CC +COG	1	1	72,828	19.41
	2	2	54,137	14.43
	3	4	98,725	26.31
	4	6	7,832	2.09
	5	8	110,082	29.34
	6	12	140,480	37.44
	7	17	50,126	13.36
C + Di + TG + RNR + CC +COG + NuR	1	1	72,828	9.29
	2	2	54,137	6.91
	3	3	70,906	9.05
	4	4	98,725	12.60
	5	5	71,586	9.13
	6	6	116,351	14.85
	7	8	110,082	14.05
	8	12	140,480	17.93
	9	17	50,126	6.40

S8. Sensitivity analysis of ground availability

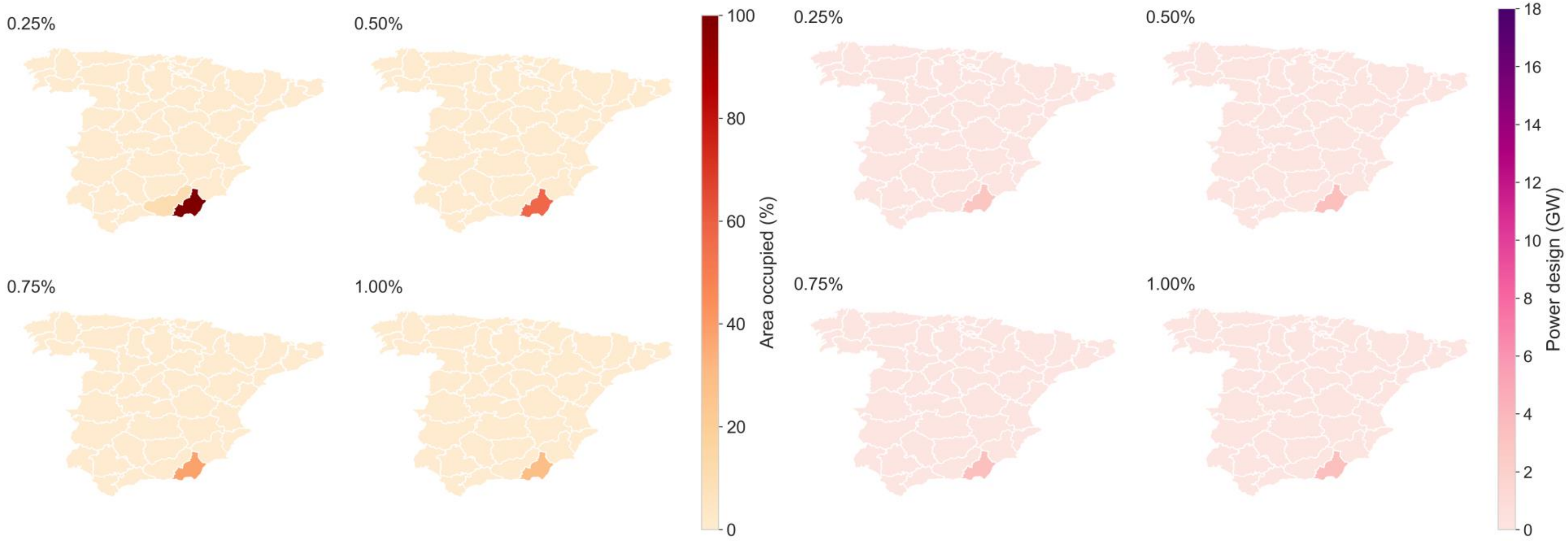

Figure S11: Effect of ground availability from 0.25% to 1.00% of total region area for Scenario A. Left: Area occupied (%); Right: power capacity installed (GW).

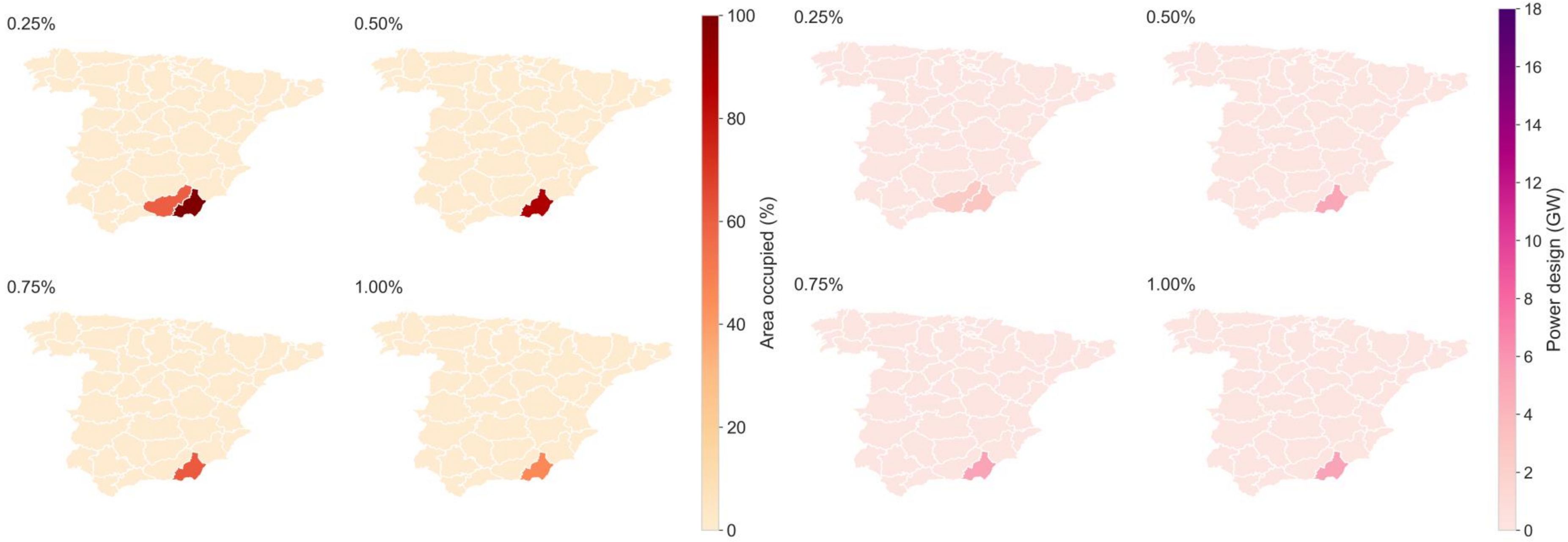

Figure S12: Effect of ground availability from 0.25% to 1.00% of total region area for Scenario B. Left: Area occupied (%); Right: power capacity installed (GW).

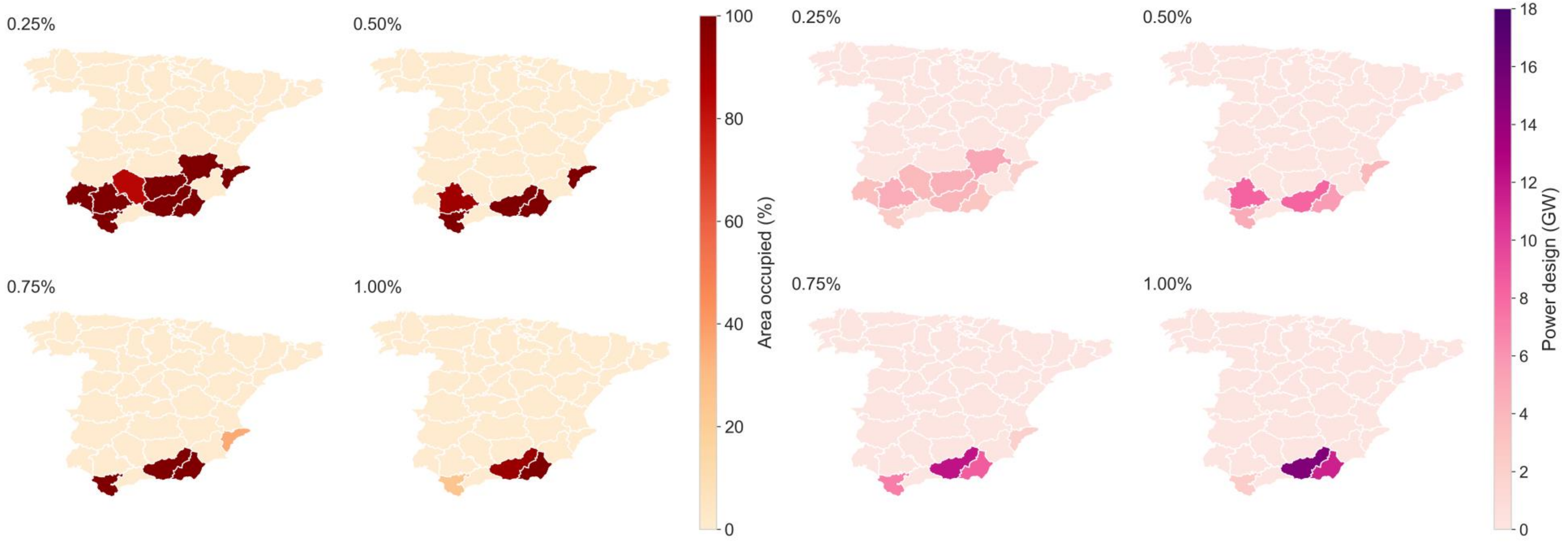

Figure S13: Effect of ground availability from 0.25% to 1.00% of total region area for Scenario C. Left: Area occupied (%); Right: power capacity installed (GW).

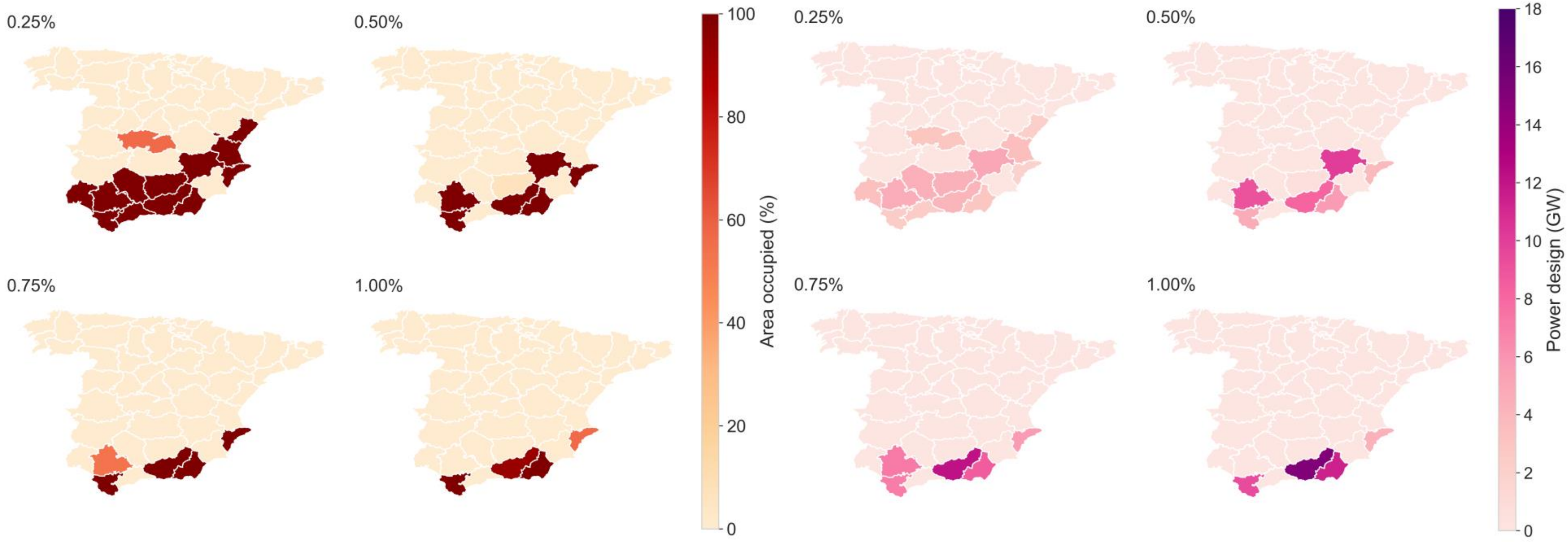

Figure S14: Effect of ground availability from 0.25% to 1.00% of total region area for Scenario D. Left: Area occupied (%); Right: power capacity installed (GW).

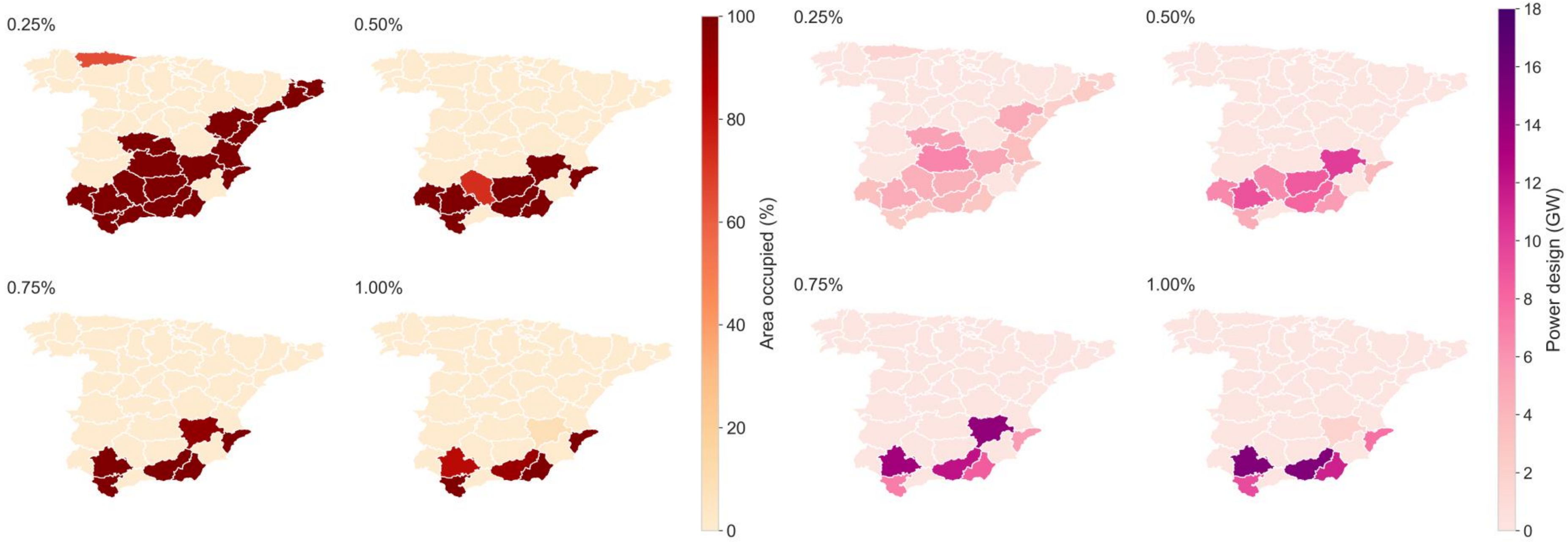

Figure S15: Effect of ground availability from 0.25% to 1.00% of total region area for Scenario E. Left: Area occupied (%); Right: power capacity installed (GW).

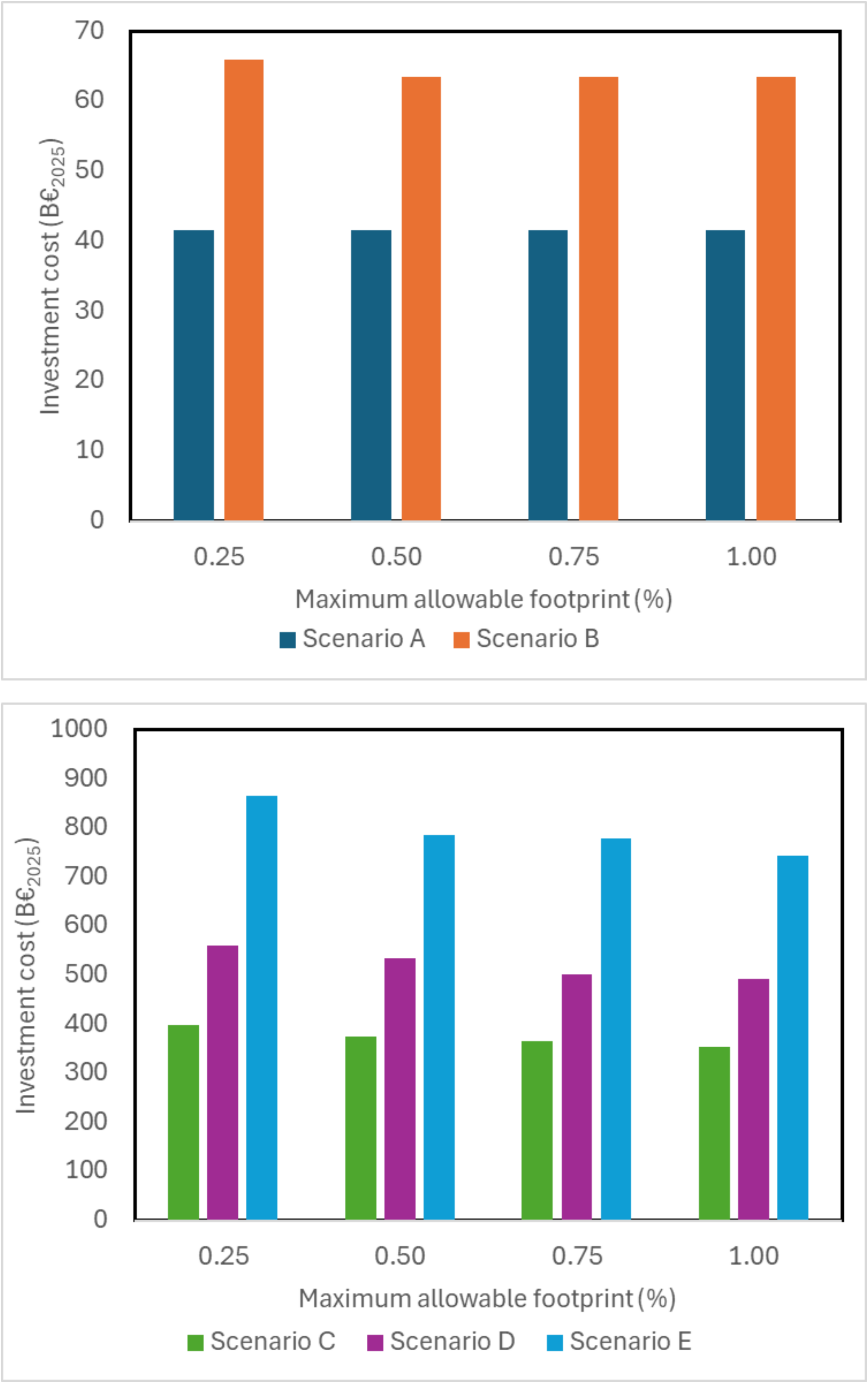

Figure S16: Effect of ground availability from 0.25% to 1.00% of total investment cost (B€$_{2025}$) for each scenario: upper section – scenarios A and B; lower section – scenarios B, C and D.

S9. Evaluation of NPV

Evaluation 1: Return ratio: 7%; Electricity selling price: 120 €/MWh.

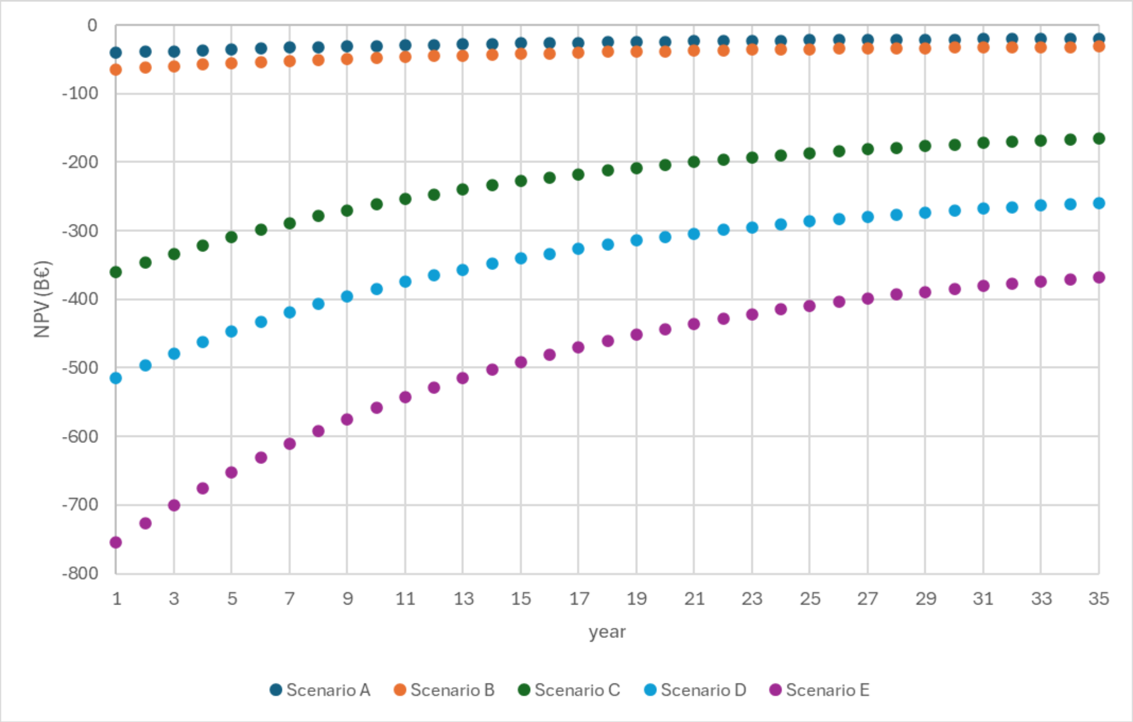

Figure S17: NPV: negative balance.

Evaluation 2: Return ratio: 3%; Electricity selling price: 136.21 €/kWh.

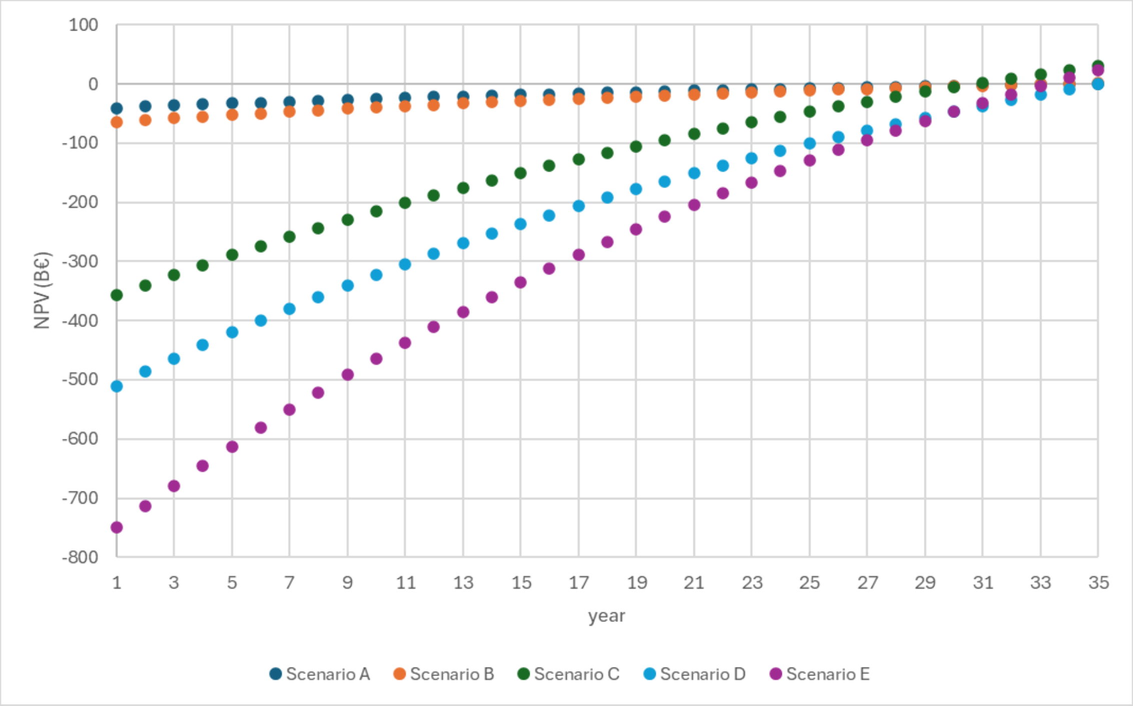

Figure S18: NPV: positive balance.

S10. Evaluation of required subsidy

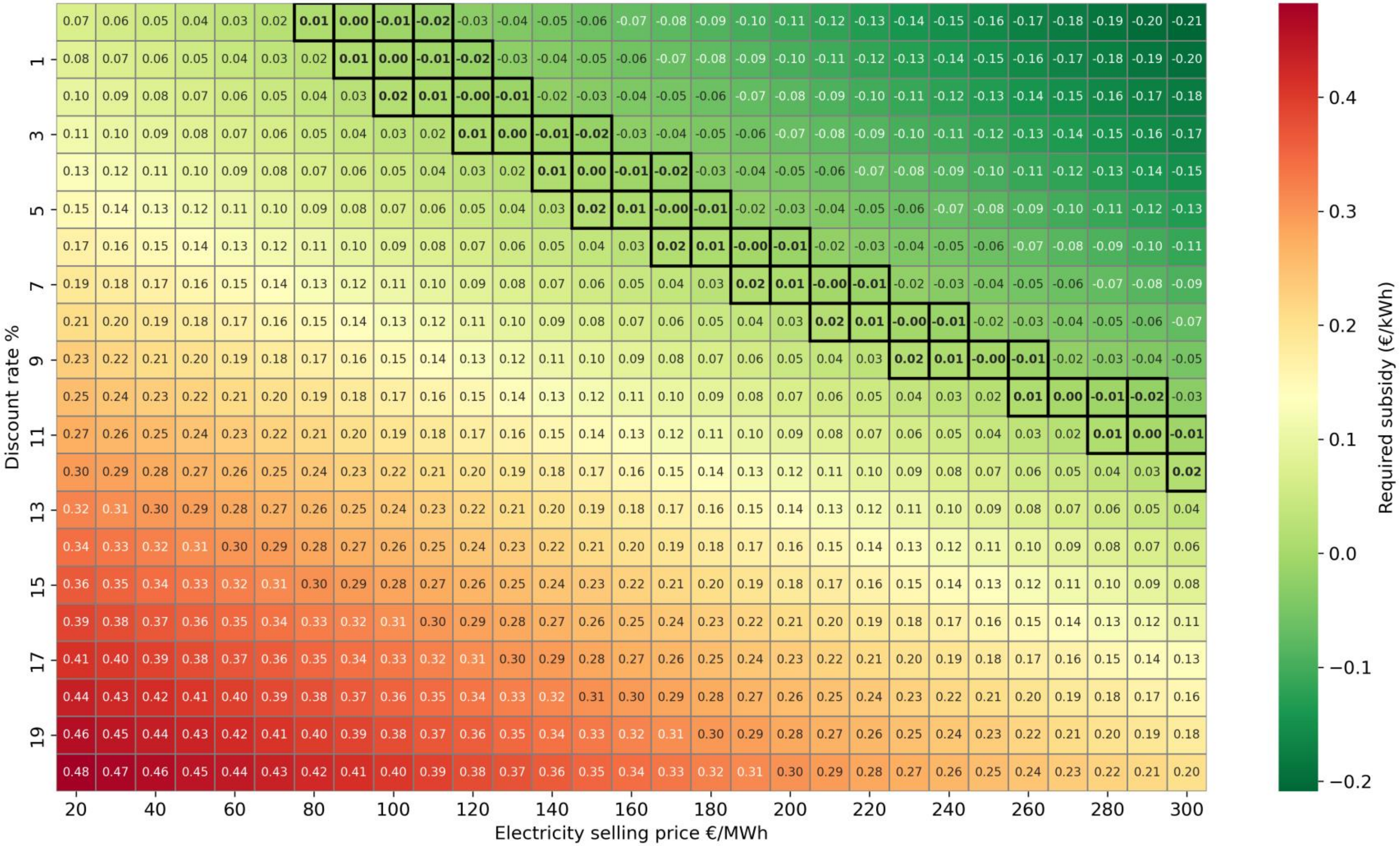

Figure S19: Subsidy required for Scenario A.

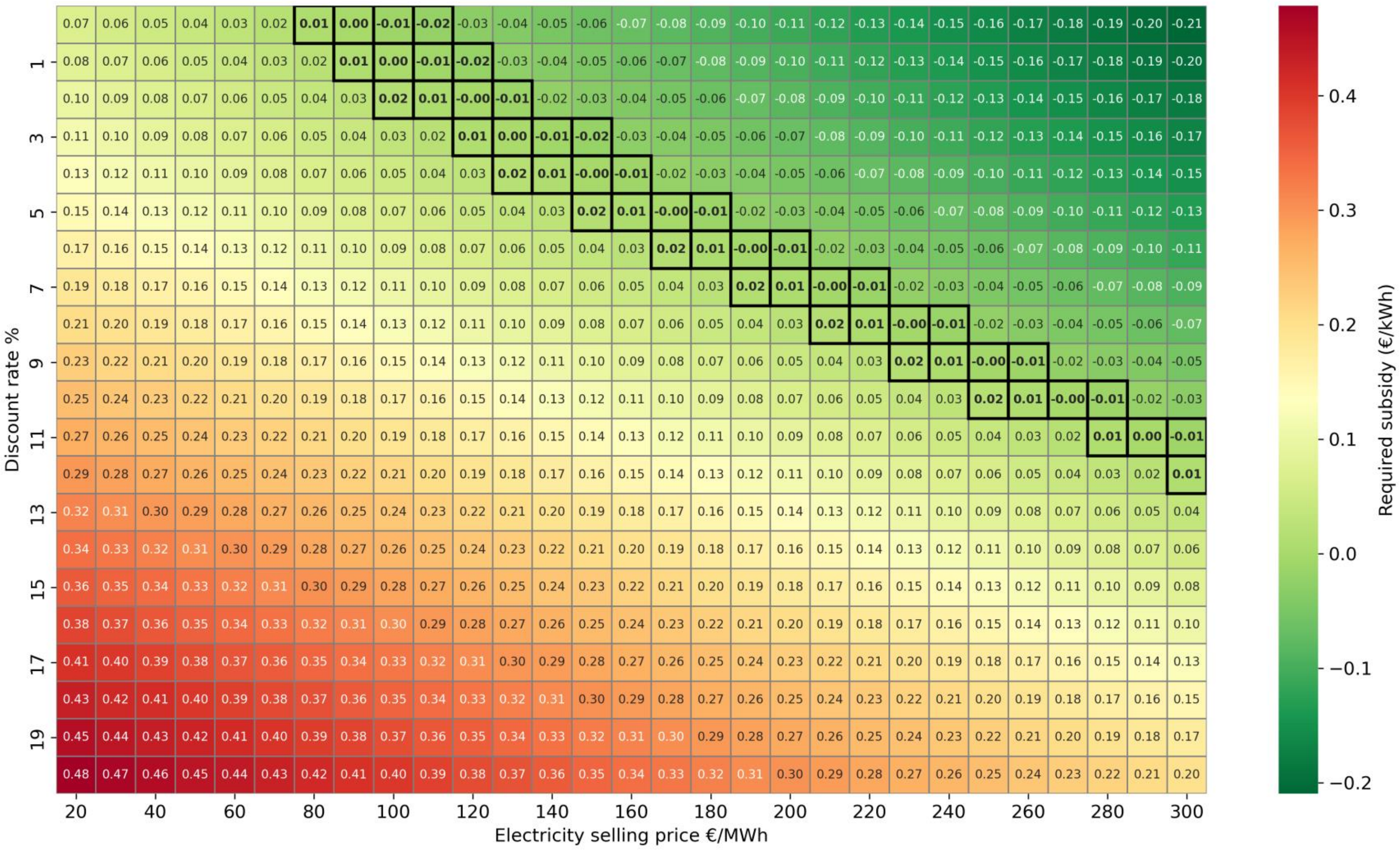

Figure S20: Subsidy required for Scenario B.

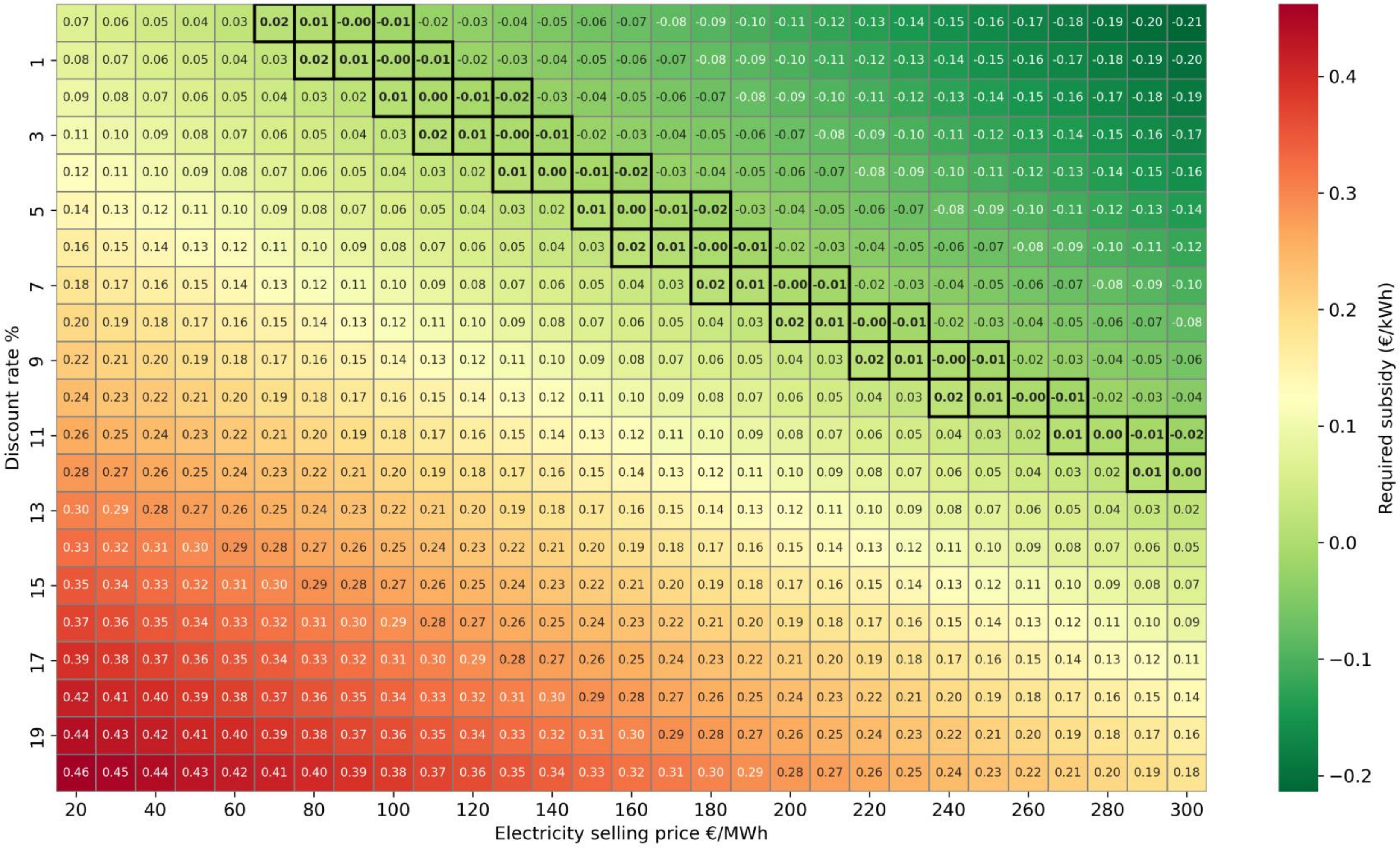

Figure S21: Subsidy required for Scenario C.

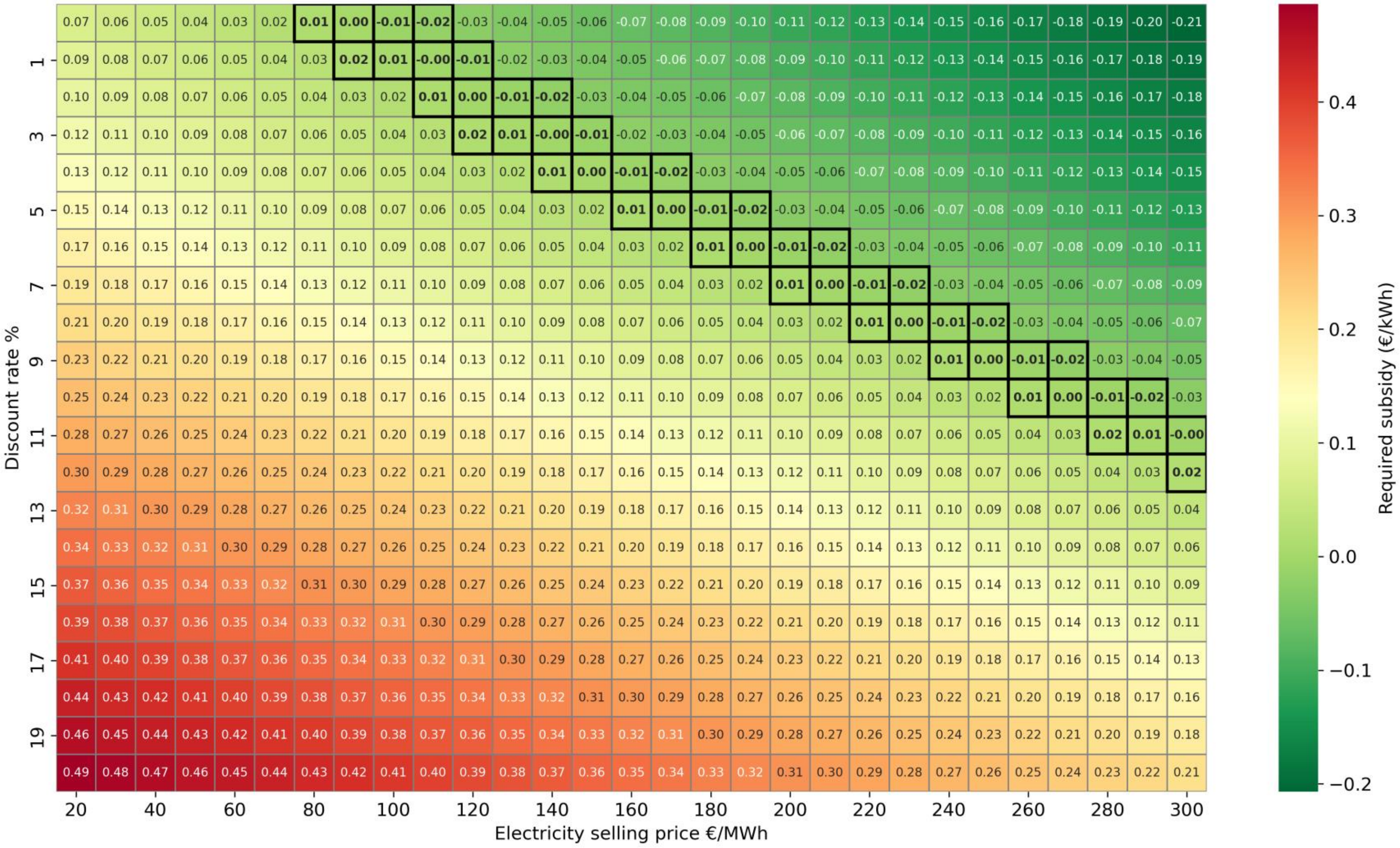

Figure S22: Subsidy required for Scenario D.

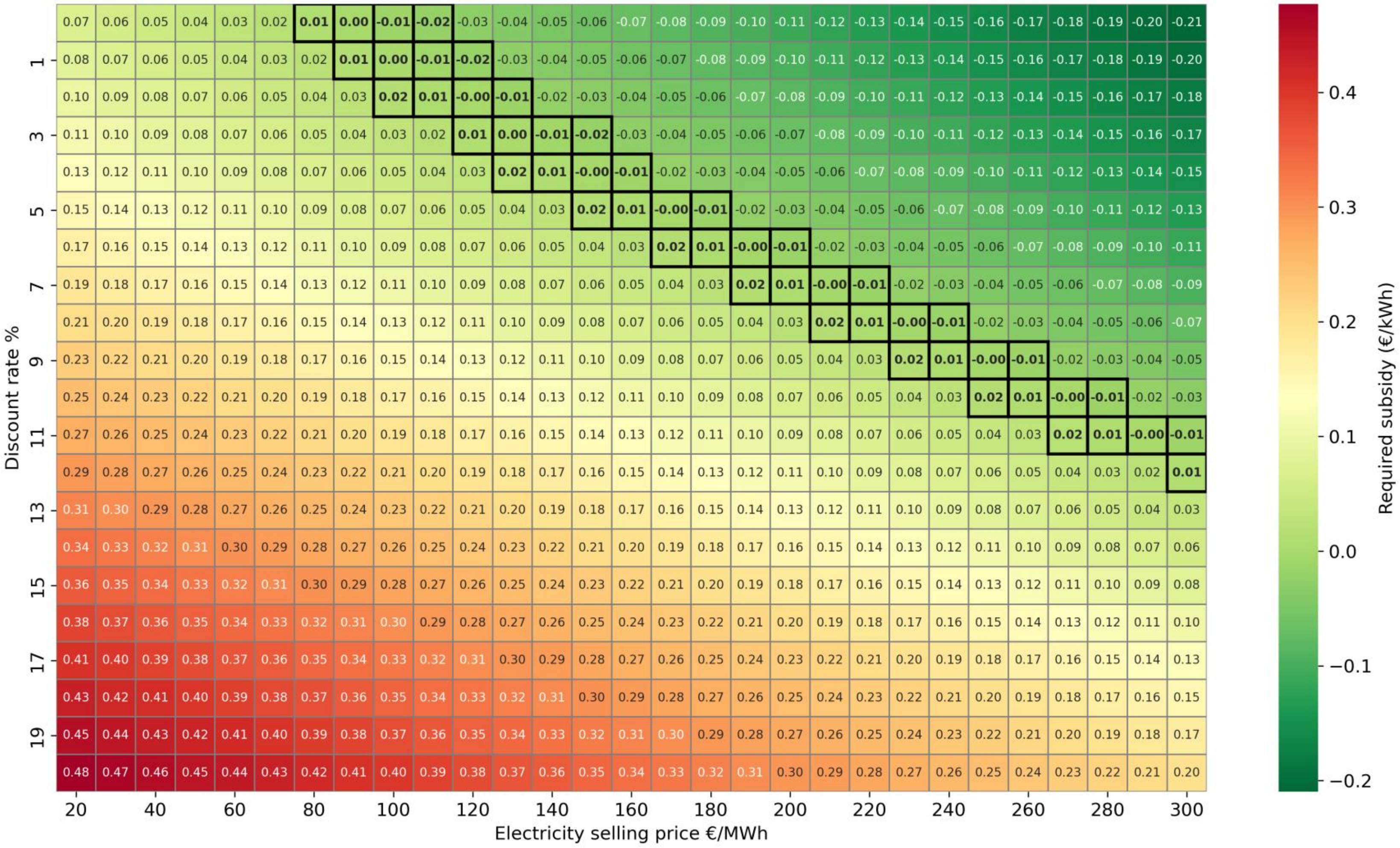

Figure S23: Subsidy required for Scenario E.

S11. Parefo front for social impact and investment cost

To obtain a Pareto-type relationship, a modified objective function was used in which all cost-related terms were grouped into one term (Z_c), and the social term was considered separately (Z_s), as seen in eq. (S107):

$$Z = P_c \cdot Z_c - P_s \cdot Z_s \tag{S107}$$

In this formulation, by varying the social weight (P_s) relative to the cost weight (P_c), different solutions can be obtained that reflect different trade-offs between total investment and social impact. To avoid unbounded investment growth, an upper limit of 1,200 B€ was imposed. The resulting Pareto-type front is shown in Figure S23:

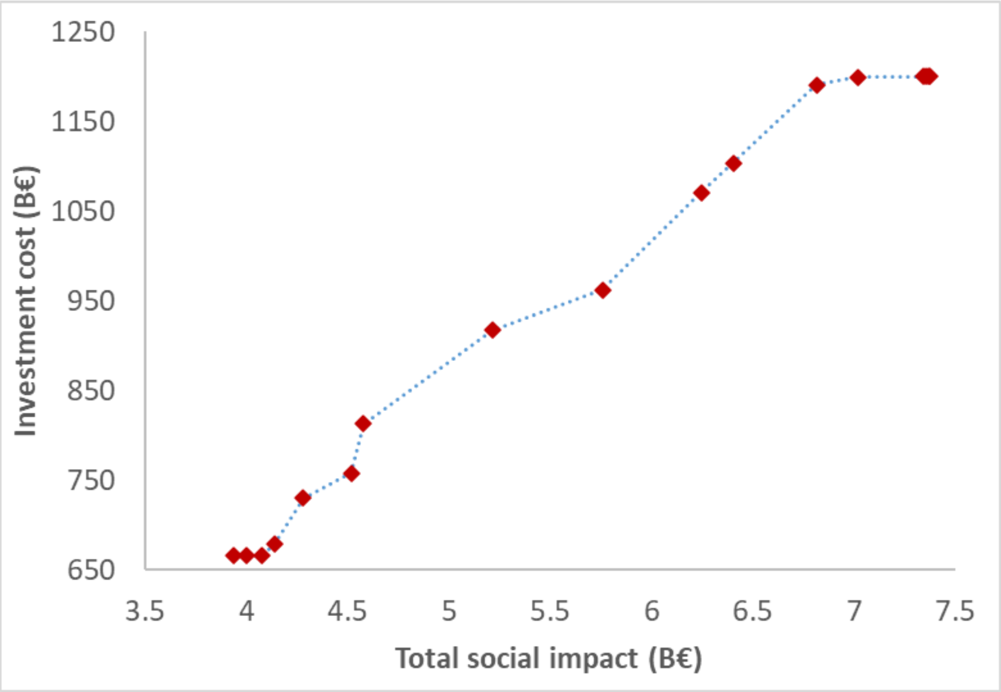

Figure S24: Pareto-type front for social impact and investment cost.

As shown in Figure S23, for social impact values around 4 B€, relatively large improvements in social impact are achieved with comparatively small increases in investment. Beyond this region, the marginal investment required to further increase social impact follows an approximately linear trend, with small oscillations arising from the discrete relocation of CSP plants as the social term gains importance. Finally, when social impact is assigned to a significantly larger weight than costs (e.g., $P_c = 1$ and $P_s = 16$ or higher), the model tends

to approach the imposed maximum investment limit, which is consistent with an optimization primarily driven by social objectives.

S12. Investment cost per capacity installed

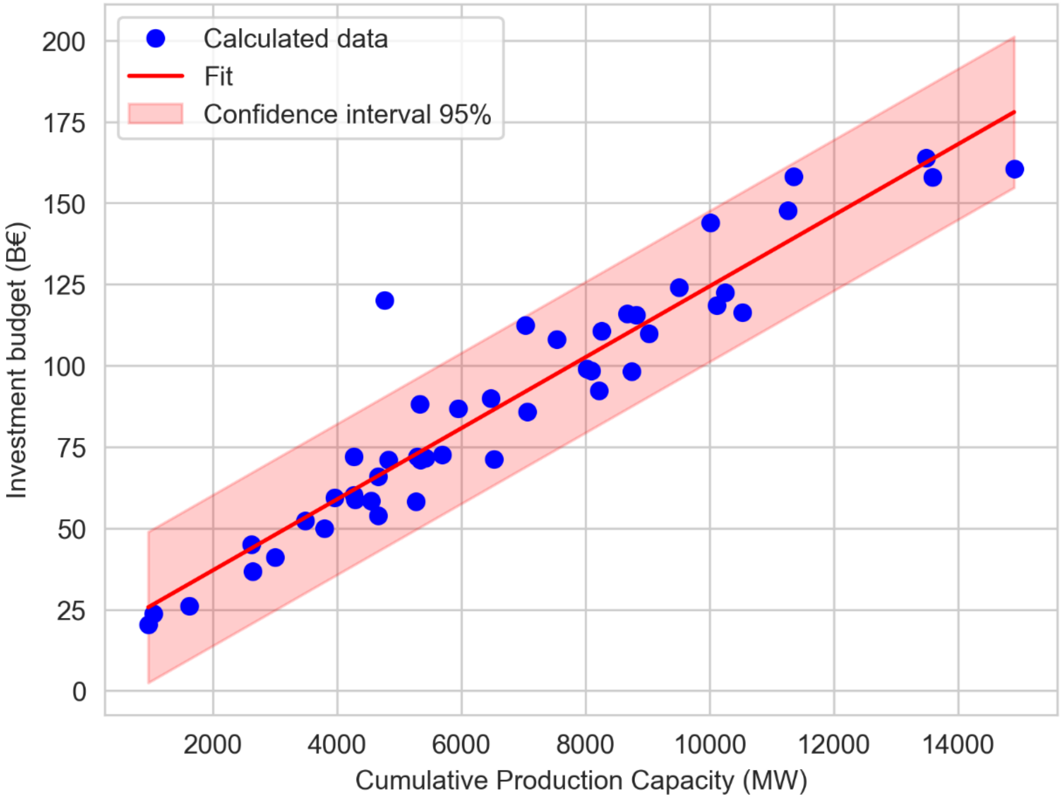

Figure S25. Investment cost (B€) per MW installed.

S13. References

[1] NREL, 2010. Concentrating Solar Power Projects. www.nrel.gov/csp/solarpaces/project_detail.cfm/projectID=40 (last accessed January 2013)

[2] R. Moore, M. Vernon, C.K. Ho, N.P. Siegel, G.J. Kolb. Design Considerations for Concentrating Solar Power Tower Systems Employing Molten Salt. (2010) SANDIA REPORT SAND2010-6978. Albuquerque, U.S.A.

[3] V. Ruiz-Hernández, La electricidad solar térmica, tan lejos, tan cerca. (2009) Fundación Gas Natural, Barcelona, Spain.

[4] T.R. Mancini, Catalog of Solar Heliostats. (2000) Tech. Rep. No. III - 1/00, IEA SolarPACES. Köln. Germany.

[5] A.B. Zavoico. Solar Power Tower Design Basis (2001) Document SAND2001-2100 San Francisco U.S.A.

[6] T.M. Pavlovic, I.S. Radonjic, D.D. Milosavljevic, L.S: Pantic A review of concentrating solar power plants in the world and their potential use in Serbia Renewable and Sustainable Energy Reviews 16 (2012) 3891–3902.

[7] P. Palenzuela, G. Zaragoza, D.C. Alarcón-Padilla, E. Guillén, M Ibarra, J. Blanco, Assessment of different configurations for combined parabolic-trough (PT) solar power and desalination plants in arid regions, Energy 36 (2011) 4950-4958.

[8] P. Palenzuela, G. Zaragoza, D.C. Alarcón-Padilla, J. Blanco, Evaluation of cooling technologies of concentrated solar power plants and their combination with desalination in the mediterranean area Applied Thermal Engineering 50 (2013) 1514-1521.

[9] C. Xu, Z. Wang, X. Li, F. Sun, Energy and exergy analysis of solar power tower plants Applied Thermal Engineering 31 (2011) 3904-3913.

[10] G.C. Bakos, N.F. Tsagas, Technical feasibility and economic viability of a small-scale grid connected solar thermal installation for electrical-energy saving. Applied Energy 72 (2002) 621–630.

[11] P. Halb, A.M. Blanco-Marigorta, B. Erlach, Exergoeconomic comparison of wet and dry cooling technologies for the Rankine cycle of a solar thermal power plant. Proceedings of ecos 2012 - the 25th international conference on efficiency, cost, optimization, simulation and environmental impact of energy systems 300-1, 300-14.

[12] G. Morin, P. Richter, P. Nitz. New method and software for multivariable technoeconomic design optimization of CSF plants. www.mathcces.rwth-aachen.de/_media/5people/richter/pascalrichter-2010-solarpaces.pdf (last accessed December 2012).

[13] P. Richter, E. Abraham, G. Morin, Optimisation of Concentrating Solar Thermal Power Plants with Neural Networks. A. Dobnikar, U. Lotric, and B. Ster (Eds.): ICANNGA 2011, Part I, LNCS 6593, pp. 190–199.

[14] A. Ghobeity, C.J. Noone, C.N. Papanicolas, A. Mitsos, Optimal time-invariant operation of a power and water cogeneration solar-thermal plant. Solar Energy 85 (2011) 2295–2320.

[15] H. Nezammahalleh, F. Farhadi, M. Tanhaemami, Conceptual design and techno-economic assessment of integrated solar combined cycle system with DSG technology. Solar Energy 84 (2010) 1696–1705.

[16] Sinnott R, Towler G. Costing and Project Evaluation. Chemical Engineering Design, Elsevier; 2020, p. 275–369. https://doi.org/10.1016/B978-0-08-102599-4.00006-0.

[17] Martín, M., Grossmann, I.E., 2022. Mathematical modeling for renewable process design, in: Sustainable Design for Renewable Processes. Elsevier, pp. 35–100. https://doi.org/10.1016/B978-0-12-824324-4.00010-X

[18] MAPA, M. de A., Pesca y Alimentación (Spanish Ministry of Agriculture, Fishing and Food), 2021. Encuesta de Precios de la Tierra 2020 (Land Price Survey 2020).

[19] Martín, L., Martín, M., 2013. Optimal year-round operation of a concentrated solar energy plant in the south of Europe. Appl. Therm. Eng. 59, 627–633. https://doi.org/10.1016/j.applthermaleng.2013.06.031

[20] Guccione, S., 2020. Design and Optimization of a SodiumMolten Salt Heat Exchanger for Concentrating Solar Power Applications (Master of Science Thesis). KTH Royal Institute of Technology, Stockholm, Sweden.

[21] Matches, 2014a. Heat Exchanger Cost Estimate. Matches. URL https://www.matche.com/equipcost/Exchanger.html (accessed 12.22.22).

[22] Camponogara, E., Nazari, L.F., 2015. Models and Algorithms for Optimal Piecewise-Linear Function Approximation. Mathematical Problems in Engineering 2015, 1–9. https://doi.org/10.1155/2015/876862

[23] Matches, 2014b. Cooling Cost Estimate. URL https://www.matche.com/equipcost/Cooling.html (accessed 12.21.22).

[24] Heras J, Martín M. Social issues in the energy transition: Effect on the design of the new power system. Appl Energy 2020;278:115654. https://doi.org/10.1016/j.apenergy.2020.115654.

[25] González-Núñez S, Guerras LS, Martín M. A multiscale analysis approach for the valorization of sludge and MSW via co-incineration. Energy 2023;263:126081. https://doi.org/10.1016/j.energy.2022.126081.

[26] INE. Tasas de actividad, paro y empleo por provincia y sexo (Activity, unemployment and employment rates by province and sex). Spanish National Institute of Statistics 2024. https://www.ine.es/jaxiT3/Tabla.htm?t=3996 (accessed January 1, 2025).

[27] INE. Cifras oficiales de población resultantes de la revisión del Padrón municipal a 1 de enero (Official population figures resulting from the revision of the municipal census as of January 1). Spanish National Institute of Statistics 2024. https://www.ine.es/jaxiT3/Tabla.htm?t=2852 (accessed January 1, 2025).

[28] INE. Contabilidad Nacional Anual de España: principales agregados. 2019-2021 (Annual National Accounts of Spain: main aggregates. 2019-2021). Spanish National Institute of Statistics 2024. https://www.ine.es/dyngs/INEbase/es/operacion.htm?c=Estadistica_C&cid=1254736177057&menu=ultiDatos&idp=1254735576581 (accessed January 1, 2025).

[29] PAHO PAHO, WHO WHO. Cantidad de agua necesaria para emergencias (Amount of water needed for emergencies). 2012.

[30] Carvajal A, Rísquez A, Echezuría L, Fernández M, Castro J, Aurentis L. Recomendaciones sobre el consumo de agua y alimentos en circunstancias especiales (Recommendations on water and food consumption in special food consumption in special circumstances). Bol venez infectol 2019;30:5–9.

[31] Embalses.net, 2025. Agua embalsada en España, año 2022 (Reservoir water in Spain, year 2022). URL https://www.embalses.net/ (accessed 12/20/2022).

[32] PVGIS, 2023. Photovoltaic Geographical Information System. URL https://re.jrc.ec.europa.eu/pvg_tools/en/ (accessed 12.12.22).

[33] NREL, 2023. Concentrating Solar Power Projects in Spain. URL https://solarpaces.nrel.gov/by-country/ES (accessed 01.16.2023).
